# Zeeman- and Orbital-Driven Phase Shifts in Planar Josephson Junctions

**DOI:** 10.1021/acsnano.3c04957

**Published:** 2023-09-11

**Authors:** Daniel Z. Haxell, Marco Coraiola, Deividas Sabonis, Manuel Hinderling, Sofieke C. ten Kate, Erik Cheah, Filip Krizek, Rüdiger Schott, Werner Wegscheider, Fabrizio Nichele

**Affiliations:** † IBM Research Europe−Zurich, 8803 Rüschlikon, Switzerland; ‡ Laboratory for Solid State Physics, ETH Zürich, 8093 Zürich, Switzerland; ¶ Institute of Physics, Czech Academy of Sciences, 162 00 Prague, Czech Republic

**Keywords:** hybrid materials, superconductor−semiconductor, phase transitions, orbital effect, spin−orbit interaction, 2DEG, φ_0_-junction

## Abstract

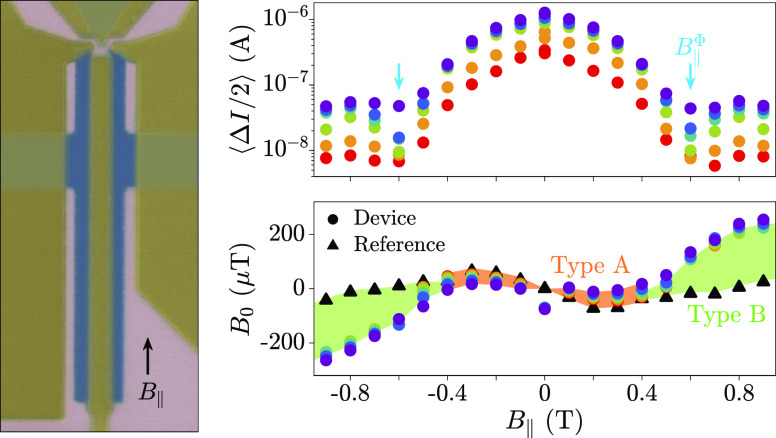

We perform supercurrent and tunneling spectroscopy measurements on gate-tunable InAs/Al Josephson junctions (JJs) in an in-plane magnetic field and report on phase shifts in the current–phase relation measured with respect to an absolute phase reference. The impact of orbital effects is investigated by studying multiple devices with different superconducting lead sizes. At low fields, we observe gate-dependent phase shifts of up to φ_0_ = 0.5π, which are consistent with a Zeeman field coupling to highly transmissive Andreev bound states via Rashba spin–orbit interaction. A distinct phase shift emerges at larger fields, concomitant with a switching current minimum and the closing and reopening of the superconducting gap. These signatures of an induced phase transition, which might resemble a topological transition, scale with the superconducting lead size, demonstrating the crucial role of orbital effects. Our results elucidate the interplay of Zeeman, spin–orbit, and orbital effects in InAs/Al JJs, giving improved understanding of phase transitions in hybrid JJs and their applications in quantum computing and superconducting electronics.

Josephson junctions (JJs) defined in hybrid superconductor–semiconductor materials are the subject of intense investigation as building blocks of gate-tunable superconducting^1−5^ and Andreev^6−18^ qubits, along with transistors,^19−22^ mixers,^23^ and rectifiers^24^ for superconducting electronics. Additional functionalities are enabled by the interplay between spin–orbit interaction and external magnetic fields, including spin-dependent^25,26^ and nonreciprocal supercurrents,^27−29^ topological phase transitions,^30−34^ and anomalous shifts in the ground state.^35−42^ The latter constitute a shift in the energy minimum away from a phase difference φ = 0 across the JJ, to 0 < φ < π by breaking of time-reversal symmetry^43−47^ or to φ = π by a Zeeman-induced phase transition.^46,48,49^

Epitaxially grown InAs/Al heterostructures^50,51^ are a promising platform to realize these complex devices, due to their high electron mobility, excellent superconducting properties,^52,53^ and prospect of scalability. Such heterostructures have a strong Rashba spin–orbit interaction,^50,54^ oriented in the plane of the electron gas and perpendicular to the wavevector of charge carriers.^55^ To date, tunneling spectroscopy experiments of planar InAs/Al JJs have revealed the onset of zero-energy states at large in-plane magnetic fields,^32,33^ and more refined devices^56^ have since shown zero-energy states accompanied by closure and reopening of the superconducting gap. While zero-energy states emerging after gap reopening were robust to changes in gate voltage, consistent with a topological phase,^30,31^ simulations have shown that these signatures could also have trivial origins.^57,58^ Supercurrent measurements in superconducting quantum interference devices (SQUIDs) demonstrated gate-tunable phase shifts in small magnetic fields,^41^ as well as large phase jumps at larger fields^34^ accompanied by a minimum in the supercurrent amplitude, also consistent with a topological transition.^30^ However, several questions remain on the behavior of planar JJs subjected to in-plane magnetic fields. For instance, ref (41) reported anomalous phase shifts at small magnetic fields which were considerably larger than theoretical expectations.^44^ Additionally, orbital effects can resemble the behavior expected from a topological transition:^30,58^ a magnetic flux threading the cross-section underneath the superconducting leads can produce non-monotonic switching currents^32,59^ together with closure and reopening of the induced superconducting gap. In this context, it is crucial to understand the mechanisms underlying phase shifts in planar JJs in an in-plane magnetic field to fully harness their properties in quantum computation and superconducting electronics applications.

In this work, we present a comprehensive investigation of planar SQUIDs in in-plane magnetic fields. An advanced device geometry allowed simultaneous measurements of the Andreev bound state (ABS) spectrum of a planar JJ and its current–phase relation (CPR), including anomalous phase shifts relative to the absolute phase reference. The role of orbital effects was studied by measuring several devices with varying sizes of the superconducting leads. For in-plane magnetic fields oriented perpendicular to the current flow in the JJ, that is along the direction of the Rashba spin–orbit field, we observed phase shifts in the CPR which depended linearly on magnetic field and varied strongly with gate voltage, similar to ref (41). For simplicity, we define this as a Type A phase shift. These gate-dependent shifts were observed over the full range of measured in-plane fields with the clearest effect at small field values. Spectroscopic measurements demonstrated that Type A phase shifts in the CPR were highly correlated with phase shifts of ballistic ABSs in the JJs, but were found to be independent of the size of the superconducting contacts. At larger magnetic fields, we observed a rapid increase in the anomalous phase shift, which occurred for all values of top-gate voltage and was strongly correlated with the length of the superconducting contacts, indicating an orbital origin. We define this as a Type B phase shift. Strikingly, Type B phase shifts were accompanied by both a local minimum in the amplitude of the CPR and a closure and reopening of the superconducting gap, which might resemble a topological transition. We discuss similarities and differences in our observations with respect to previous work. Our results establish a baseline understanding of InAs/Al JJs subject to in-plane magnetic fields, including anomalous phase shifts and topological transitions in planar JJs.

## Results

**Figure 1:**
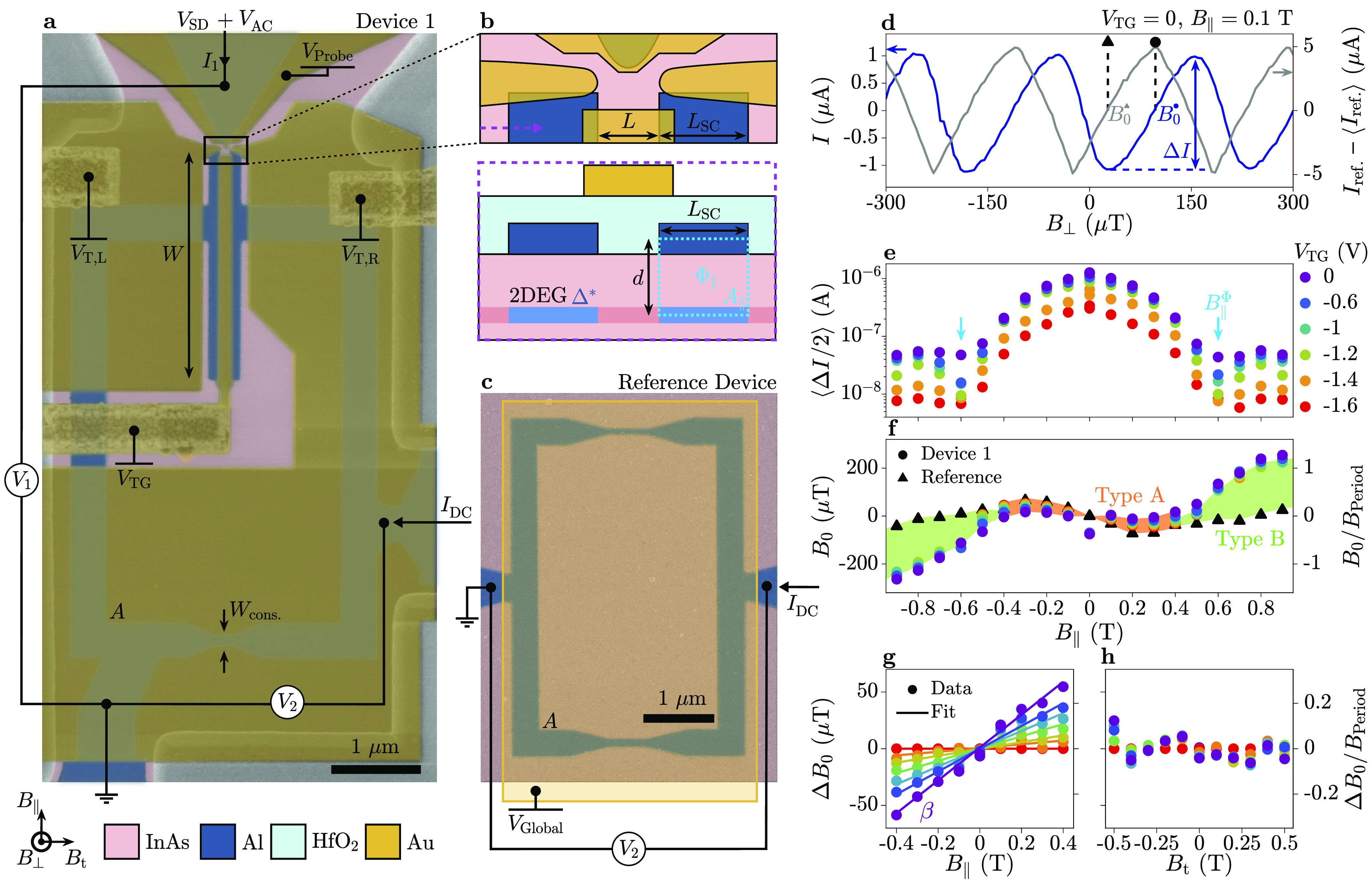
Device under study and current-biased measurements in an in-plane magnetic field *B*_∥_. (a) False-colored scanning electron micrograph (SEM) of Device 1, the planar superconducting quantum interference device (SQUID), consisting of InAs (pink) and Al (blue). Exposed InAs regions were controlled via electrostatic gates (yellow). (b) Schematic zoom-in of the Josephson junction region (top), with junction length *L* = 80 nm and superconducting lead length *L*_SC_ = 250 nm indicated. The purple dashed line indicates the position of a schematic cross-section (bottom). An in-plane magnetic field *B*_∥_ generates a flux Φ_∥_ between the superconducting leads and the proximitized two-dimensional electron gas (2DEG), with area *A*_∥_ = *L*_SC_*d*. (c) False-colored SEM of the reference device, prior to gate deposition, consisting of two Al constrictions embedded in a superconducting loop. A global gate *V*_Global_ is indicated schematically (yellow). (d) Switching current *I* of Device 1 as a function of perpendicular magnetic field *B*_⊥_ (blue), at a top-gate voltage *V*_TG_ = 0 and an in-plane magnetic field *B*_∥_ = 0.1 T, after removing a background of 37 μA corresponding to the Al constriction. Switching current of the reference device *I*_ref._ (gray) at the same *B*_∥_, after subtracting the average ⟨*I*_ref._⟩. The zero-current position for Device 1 (reference device) is indicated by the circle (triangle). (e) Averaged half-amplitude of a SQUID oscillation ⟨Δ*I*/2⟩ as a function of the in-plane magnetic field *B*_∥_, for different top-gate voltages *V*_TG_ (colors). A minimum in ⟨Δ*I*/2⟩ occurred at *B*_∥_ = *B*_∥_^Φ^ (turquoise arrows). (f) Shift in the perpendicular magnetic field *B*_0_ of Device 1 (circles) and reference device (triangles), as a function of *B*_∥_. Deviation of Device 1 from the reference device is highlighted in orange for |*B*_∥_| ≲ 0.4 T and in green for |*B*_∥_| ≳ 0.4 T. (g) Perpendicular field shift Δ*B*_0_ for small *B*_∥_ for each *V*_TG_ (circles), with a linear fit (lines) of gradient β. Data are plotted relative to *V*_TG_ = −1.6 V. (h) Perpendicular field shift Δ*B*_0_ for in-plane field *B*_t_ applied along the transverse direction.

Experiments were performed on six devices. Figure 1(a) shows a false-colored scanning electron micrograph of Device 1, the principal device under study, which consisted of a planar SQUID fabricated in a heterostructure of InAs (pink) and epitaxial Al (blue).^50,51^ The device was covered by a HfO_2_ dielectric layer onto which Au gate electrodes (yellow) were deposited. The superconducting loop, defined in the epitaxial Al, contained a superconductor–normal semiconductor–superconductor (SNS) JJ and a narrow Al constriction. The SNS junction had a length *L* = 80 nm, width *W* = 2.5 μm, and Al leads of length *L*_SC_ = 250 nm. Similar to previous work,^32−34,56^ the junction width was chosen to limit hybridization of topological states potentially emerging at the junction ends.^30,31^ The constriction had a width *W*_cons._ = 130 nm, chosen to limit the switching current of the planar SQUID, while still being much larger than that of the SNS junction. The constriction was 500 nm long, to clearly define the narrow region of the Al, for both improved control in fabrication and consistency in average switching currents. The asymmetric SQUID configuration resulted in a phase drop across the SNS junction of φ ≈ 2π(Φ/Φ_0_), where a flux Φ = *AB*_⊥_ threaded the area *A* = 10.2 (μm)^2^ enclosed by the SQUID loop (Φ_0_ = *h*/2*e* is the superconducting flux quantum). This gave an oscillating switching current that was periodic in perpendicular magnetic field *B*_⊥_, with period *B*_Period_ = 200 μT. Differently from previous work,^32,34,41,53^ where two InAs JJs were used, the Al constriction cannot introduce anomalous phase shifts in an in-plane magnetic field due to the absence of spin–orbit and orbital effects. A superconducting probe was integrated close to one end of the SNS junction, comprising a contact of epitaxial Al separated from the SNS junction by a tunnel barrier defined in the InAs. The transparency of the tunnel barrier was controlled by the gate voltages *V*_T,L_ and *V*_T,R_, applied to the left and right tunnel gates, respectively. The carrier density in the SNS junction was controlled via top-gate voltage *V*_TG_. An additional gate was kept at *V*_Probe_ = 0 throughout. Devices 2 to 5 were similar to Device 1 except for *L*_SC_, resulting in different orbital coupling to in-plane magnetic fields [see Figure 1(b)]. Switching current measurements were performed on Devices 1 to 4, with complementary tunneling spectroscopy measurements carried out on Devices 1 and 5 (see Supporting Information, Sections 6–8). Each switching current measurement presented here was acquired in parallel with measurements of a reference device fabricated on the same chip, which consisted of a SQUID with two Al constrictions of different widths enclosing an area *A* [see Figure 1(c)]. The oscillation periods of Devices 1 to 5 and the reference device were similar (all within 9 μT, corresponding to 5% of *B*_Period_). Parallel conduction in the InAs surrounding the reference devices was prevented by setting a global gate to *V*_Global_ = −3 V, such that the switching current of the reference device was independent of *V*_Global_. Further, no *V*_Global_-dependent phase shifts were observed in the reference device at elevated in-plane magnetic fields, showing the absence of spin–orbit effects (see Supporting Information, Figure S.1, for more details).

Switching currents *I* were measured by using fast current ramps and voltage triggers. A ramped current *I*_DC_ was injected into the SQUID loop while the voltage *V*_2_ across the device was monitored with an oscilloscope. The switching current was defined as the value of *I*_DC_ at which *V*_2_ exceeded a threshold set to 15% of the maximum voltage in the resistive state. Particular care was taken to inject the current *I*_DC_ by symmetrically biasing the measurement circuit, to prevent significant voltage buildup between SQUID and gates. Each CPR data point shown here was obtained by averaging over 32 data points measured with *I*_DC_ > 0 and 32 with *I*_DC_ < 0. This procedure allowed us to improve the experimental accuracy, limit the effect of the broad switching current distributions typical of planar devices,^60^ and cancel trivial phase shifts originating from the kinetic inductance of the loop.^61^ The CPR of the SNS junction was obtained by subtracting the switching current of the Al constriction *I*_Al_ from that of the SQUID loop, which had a value between 30 and 45 μA for all devices. Tunneling conductance measurements were performed by low-frequency lock-in techniques. A voltage bias *V*_SD_ + *V*_AC_ was sourced at the tunneling probe, and the resulting AC current *I*_1_ and voltage *V*_1_ gave the differential conductance *G* ≡ *I*_1_/*V*_1_. Global magnetic fields were applied via a three-axis vector magnet, nominally along the directions *B*_⊥_, *B*_∥_, and *B*_t_ as indicated in Figure 1(a). Further details on electronic measurements and on the procedures used to accurately align the chip to the external magnetic field are, respectively, presented in the Methods and Supporting Information (Section 3).

Figure 1(d) shows the CPR of Device 1 at *V*_TG_ = 0 (blue line, left axis) and the reference device (gray line, right axis) at *B*_∥_ = 0.1 T. We highlight the maximum switching current Δ*I*/2 and a *B*_⊥_-field shift *B*_0_, which was measured where the CPR crossed zero with a positive slope (circle and triangle for Device 1 and reference device, respectively). Figure 1(e) and (f) show Δ*I*/2 and *B*_0_, respectively, as functions of *B*_∥_ and for various values of *V*_TG_. Black triangles in Figure 1(e) represent magnetic field shifts measured in the reference device. In Figure 1(e) we plot ⟨Δ*I*/2⟩, that is, the maximum supercurrent Δ*I*/2 averaged over positive and negative *I*_DC_. We observe a non-monotonic dependence of ⟨Δ*I*/2⟩ as a function of *B*_∥_, with minima at *B*_∥_ = ± *B*_∥_^Φ^ = ±0.6 T (see turquoise arrow) independent of the top-gate voltage. The magnetic field shift *B*_0_ in Figure 1(f) shows two distinctive trends. For |*B*_∥_| ≲ 0.4 T, *B*_0_ shows a gate-dependent deviation with respect to the reference device (Type A shift, orange shaded area). Type A shifts were larger for *V*_TG_ = 0 (purple) than for *V*_TG_ = −1.6 V (red). For |*B*_∥_| ≳ 0.4 T we observe a more pronounced shift away from the reference device (Type B shift, green shading) for all values of top-gate voltage. Notably, at *B*_∥_ = ± *B*_∥_^Φ^, where the supercurrent was at a minimum, the shift was approximately half a SQUID period, corresponding to a phase shift of ∼±π. At *B*_∥_ = 0.9 T, the magnetic field shift accumulated in Device 1 exceeded one SQUID period. Measurements at larger *B*_∥_ were not possible, due to a large reduction in switching current attributed to a portion of the Al constriction becoming resistive (see Supporting Information, Figure S.2). Gate-dependent phase shifts were observed for all values of the in-plane field *B*_∥_, consistent with Type A shifts extending over the full range of *B*_∥_. In contrast, Type B shifts occurred at large *B*_∥_ and had a similar size for all values of the gate voltage. Finally, we note a weak “S”-shaped dependence of *B*_0_, for both Device 1 and the reference device, which persisted after accurate alignment of the external magnetic field to within one oscillation period over the full range of *B*_∥_ (see Supporting Information, Section 1). We speculate that the residual trend in *B*_0_ originated from flux focusing^62^ or a nonlinearity of the vector magnet.

Geometry-dependent effects at small in-plane magnetic fields require phase shifts to be compared in the same device. We therefore quantify Type A shifts at small *B*_∥_ relative to the most negative top-gate voltage, *V*_TG_ = −1.6 V, where the spin–orbit coupling strength is assumed to be small.^41,54^Figure 1(g) shows Δ*B*_0_, that is, *B*_0_ as in Figure 1(f) after subtraction of the data at *V*_TG_ = −1.6 V, in the range of *B*_∥_ where only Type A shifts were present. At each gate voltage, the field shift (circles) was approximately linear in *B*_∥_, as highlighted by the linear fits (solid lines). The slope β extracted from the linear fits increased for more positive *V*_TG_. Remarkably, no significant phase shift of either Type A or B was observed for in-plane fields *B*_t_ applied along the transverse direction, as shown in Figure 1(h) for Type A shifts (see Supporting Information, Section 4, for further details). The lack of Type A shifts as a function of *B*_t_ implies a direction-dependent coupling to the external field with a coupling strength indicated by β.

**Figure 2:**
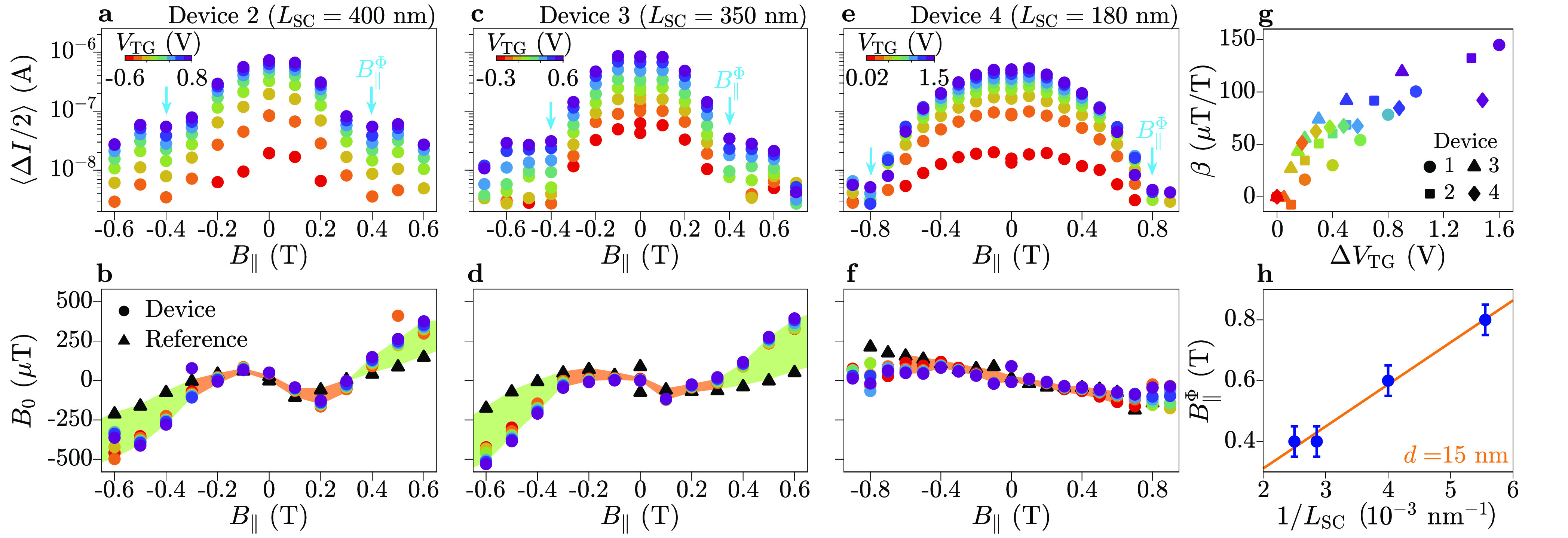
Switching current and perpendicular magnetic field shift for devices with varying *L*_SC_. (a) Average oscillation amplitude ⟨Δ*I*/2⟩ of Device 2: a planar superconducting quantum interference device (SQUID) with a superconducting lead length of *L*_SC_ = 400 nm as a function of in-plane magnetic field *B*_∥_ for different top-gate voltages *V*_TG_ (colors). Minima in the oscillation amplitude, *B*_∥_^Φ^, are marked with blue arrows. (b) Shift in perpendicular magnetic field, *B*_0_, of Device 2 (circles) and the reference device (triangles), as a function of *B*_∥_. Deviation of Device 2 from the reference device is highlighted in orange for small *B*_∥_ and in green for large *B*_∥_. (c, d) and (e, f) are the same as (a, b) for Devices 3 and 4, respectively. All devices are identical in design other than the length of the superconducting contacts, which is *L*_SC_ = 350 nm for Device 3 and *L*_SC_ = 180 nm for Device 4. (g) Gradient β of Type A phase shifts at small *B*_∥_, for Devices 1 to 4 (circles, squares, triangles, and diamonds, respectively), plotted against the change in top-gate voltage Δ*V*_TG_ with respect to the minimum value. (h) In-plane magnetic field where the supercurrent is minimum, *B*_∥_^Φ^, as a function of inverse superconducting lead length 1/*L*_SC_ (blue circles), with a linear fit *B*_∥_^Φ^ = (Φ_0_/*d*)/*L*_SC_ (orange line) giving *d* = 15 nm.

We now present CPR data obtained from Devices 2, 3, and 4, where *L*_SC_ was 400, 350, and 180 nm, respectively. Switching currents Δ*I*/2 are shown in Figure 2(a, c, e) for Devices 2–4, respectively, with field shifts *B*_0_ in Figure 2(b, d, f) for each device (colored markers) alongside those of a reference device measured in parallel (black triangles). Devices 2, 3, and 4 showed a qualitatively similar behavior to Device 1, despite having *B*_∥_^Φ^ = 0.4 T, *B*_∥_^Φ^ = 0.4 T, and *B*_∥_^Φ^ = 0.8 T, respectively. We repeated the analysis on Type A phase shifts presented in Figure 1(g) on the data of Figure 2(b, d, f) and show the extracted β in Figure 2(g) (see Supporting Information, Section 10, for more details). As each device operated in a different range of *V*_TG_, we compare them by plotting β as a function of Δ*V*_TG_, the top-gate voltage relative to the most negative value at which oscillations were observed. Despite some scattering for small Δ*V*_TG_, where data analysis is intricate due to the small switching current, we note that β follows a similar trend in all devices. In particular, β increases with Δ*V*_TG_ and does not depend on *L*_SC_. Figure 2(h) shows *B*_∥_^Φ^ as a function of the inverse superconducting lead length 1/*L*_SC_. The data (blue circles) followed a linear trend, fitted by *B*_∥_^Φ^ = (Φ_0_/*d*)/*L*_SC_ (orange line) describing one flux quantum threading an area *L*_SC_*d*. The result of *d* = 15 nm agrees with the separation of Al and InAs layers, indicating a crucial role of orbital effects in inducing Type B phase shifts.

**Figure 3:**
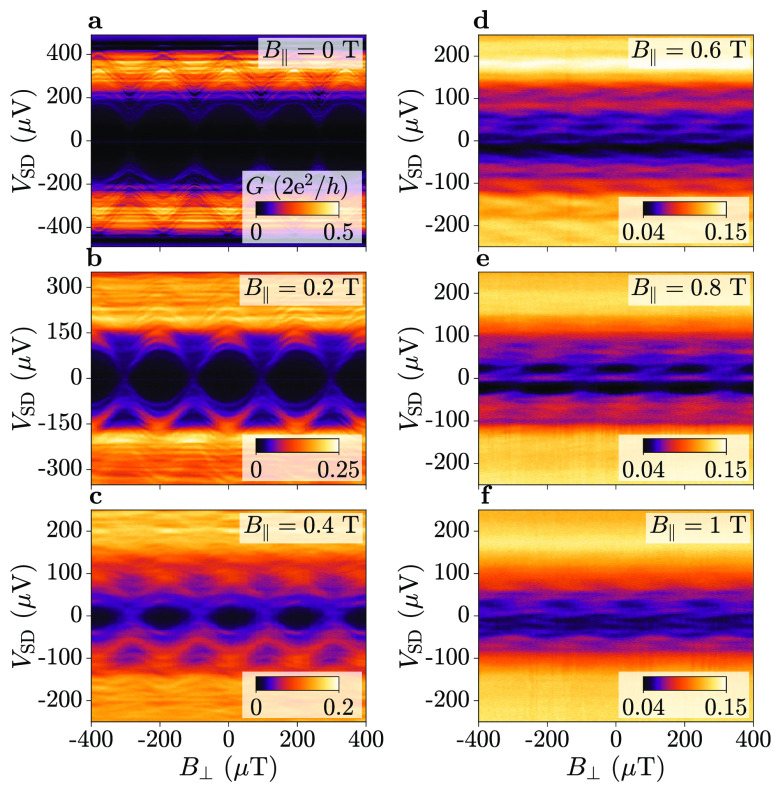
Tunneling spectroscopy of Andreev bound states in Device 1, as a function of the in-plane magnetic field *B*_∥_. (a–f) Differential conductance *G* through the tunneling probe, as a function of source–drain bias voltage *V*_SD_ and perpendicular magnetic field *B*_⊥_, for increasing values of *B*_∥_. Measurements were taken at a top-gate voltage of *V*_TG_ = −1 V, with tunnel-barrier voltages (*V*_T,L_, *V*_T,R_) = (−1.495, −1.65) V.

We now complement CPR measurements with spectroscopic data obtained on Device 1. Figure 3 presents a series of differential conductance maps as functions of *B*_⊥_ and *V*_SD_, for increasing values of *B*_∥_. All data were obtained at *V*_TG_ = −1 V (data at more values of *V*_TG_ are reported in Section 7 of the Supporting Information). As the tunneling probe was constituted by a superconducting lead, the differential conductance *G* at *B*_∥_ = 0 indicates the density of states in the junction up to a bias shift of ±*e*Δ. Further conductance peaks at zero and high bias are attributed to a residual supercurrent and multiple Andreev reflections through the tunneling probe, respectively. For *B*_∥_ ≤ 0.2 T, the conductance demonstrates a conventional spectrum containing multiple ABSs, some of which have transmission approaching unity and an induced superconducting gap of approximately 180 μeV. For *B*_∥_ ≥ 0.2 T, a finite density of states at the Fermi level was induced in the lead facing the tunneling probe, resulting in a direct mapping of the density of states in the junction.^62^ For *B*_∥_ = 0.4 T, phase-dependent conductance features approached zero energy, resulting in a significant decrease in the superconducting gap [Figure 3(c)]. For *B*_∥_ = *B*_∥_^Φ^ = 0.6 T [Figure 3(d)], conductance features close to *V*_SD_ = 0 had weak dependence on the perpendicular magnetic field and showed no clear separation from features at higher bias. We consider these features to signal a closure of the superconducting gap within 0.1 T of *B*_∥_^Φ^. As *B*_∥_ was further increased, a gap reopened in the ABS spectrum with discrete states around zero energy. Finally, the gap closed for *B*_∥_ ≥ 1 T. Conductance features close to *V*_SD_ = 0 in Figure 3(e) were reminiscent of zero-bias peaks reported for similar devices at high in-plane magnetic fields and understood in terms on topological states.^32,33^ However, unlike in previous work, zero-bias features of Figure 3(e) were not robust to small changes in the top-gate voltage *V*_TG_ or tunnel gate voltage *V*_T_ (Supporting Information, Section 5). Tunneling spectroscopy measurements of Device 5 (with *L*_SC_ = 400 nm, identical to that in Device 2) showed the closure of the superconducting gap at *B*_∥_ = 0.4 T (see Supporting Information, Section 8). This was consistent with the *B*_∥_^Φ^ value in Device 2, where the switching current reached a minimum [Figure 2(a)], giving further evidence that orbital effects have a role in suppressing the proximitized superconducting gap.

**Figure 4:**
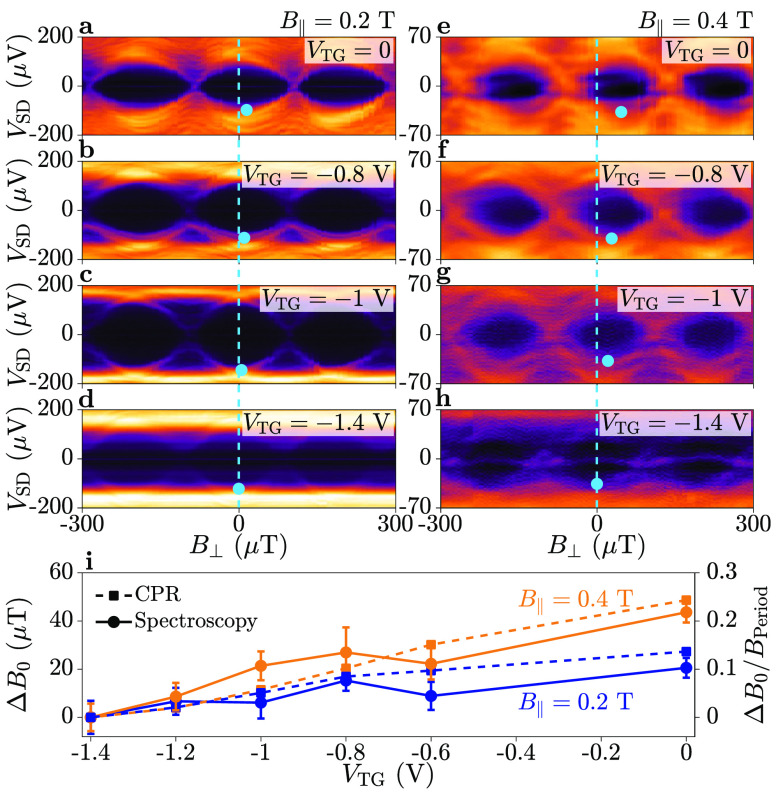
Top-gate dependence of the energy minimum at a finite in-plane magnetic field, *B*_∥_, in Device 1. (a–d) Differential conductance *G* as a function of bias *V*_SD_ and perpendicular magnetic field *B*_⊥_, at an in-plane magnetic field of *B*_∥_ = 0.2 T. Spectroscopy was performed at a top-gate voltage of *V*_TG_ = {0, −0.8, −1, −1.4} V, respectively. The blue dashed line indicates the energy minimum at *V*_TG_ = −1.4 V. Blue markers show the shift of the energy minimum as a function of *V*_TG_ relative to *V*_TG_ = −1.4 V. (e–h) Bias-dependent spectroscopy as in (a–d) at an in-plane magnetic field of *B*_∥_ = 0.4 T. (i) Shift in perpendicular magnetic field Δ*B*_0_ relative to *V*_TG_ = −1.4 V, at an in-plane magnetic field of *B*_∥_ = 0.2 T (blue) and *B*_∥_ = 0.4 T (orange), obtained from tunneling spectroscopy (circles, solid lines) and current–phase relation (CPR) measurements (squares, dashed lines). The phase shift φ_0_/2π ≡ Δ*B*_0_/*B*_Period_ is plotted on the right axis.

Figure 4 compares spectroscopic maps obtained at *B*_∥_ = 0.2 T (a–d) and 0.4 T (e–h), for multiple values of *V*_TG_. The value of *B*_⊥_ at which the ABS energy was closest to the gap was found for each value of *V*_TG_, as indicated by the blue circles. This was determined as the *B*_⊥_ value where the gradient ∂*G*/∂*B*_⊥_ was zero at a fixed bias *V*_SD_ and averaged over multiple periods. Blue dashed lines indicate the minimum energy position at *V*_TG_ = −1.4 V, which is defined as *B*_⊥_ = 0 in Figure 4(d). For both *B*_∥_ = 0.2 and 0.4 T, a clear deviation of the ABS spectrum took place as a function of *V*_TG_. The shift in perpendicular field Δ*B*_0_ measured from the ABS spectrum is summarized in Figure 4(i) as a function of *V*_TG_ for *B*_∥_ = 0.2 T (blue) and *B*_∥_ = 0.4 T (orange). The Type A shift Δ*B*_0_ obtained from the CPR is plotted on the same axis (squares, dashed lines) and shows remarkable agreement.

## Discussion

After demonstrating the occurrence of two types of anomalous phase shifts taking place in hybrid SQUIDs in in-plane magnetic fields, we now discuss their origin. Type A phase shifts, which were approximately linear in *B*_∥_ and depended on *V*_TG_ [Figure 1(g)], are associated with spin–orbit-induced anomalous phase shifts,^43−47^ as recently reported in similar devices.^41^ As phase shifts were much more pronounced for in-plane fields aligned perpendicular to the current flow direction (*B*_∥_) than parallel to it (*B*_t_) [Figure 1(h)] and were stronger for higher electron density (more positive *V*_TG_(54)), we conclude that spin–orbit interaction in our samples is predominantly of Rashba type.

Type A phase shifts reported here, which are of similar size to those in ref (41), are orders of magnitude larger than theoretical predictions.^44,46,63^ Reference (41) proposed that the observed phase offsets could be explained by the contribution of several low-transmission modes. However, here we show that Type A shifts obtained from the CPR matched those from tunneling spectroscopy (Figure 4), where conductance features at both high and low bias showed a phase shift. Since conductance features at low bias correspond to ABSs with high transmission, we conclude that highly transmissive modes participate in the overall phase shift despite their large Fermi velocity. While this result does not resolve the discrepancy between theoretical predictions and experiments,^41^ it rules out diffusive modes with small Fermi velocities as the dominant cause of Type A phase shifts.

Type B phase shifts were concomitant with a reentrant supercurrrent and a closure and reopening of the superconducting gap for all values of top-gate voltage *V*_TG_. At *B*_∥_ = ±*B*_∥_^Φ^, where the supercurrent was at a minimum and the proximitized superconducting gap was suppressed, the phase shift was φ_0_ ≈ ±π. For |*B*_∥_| > *B*_∥_^Φ^, a gap reopened in the ABS spectrum, and the phase shift increased to above 2π. A phase shift occurring with a supercurrent minimum and gap closure indicates a 0−π transition at *B*_∥_ = *B*_∥_^Φ^, where the minimum ABS energy moves from φ ≈ 0 to φ ≈ π due to coupling of the magnetic and superconducting orders by Zeeman interaction.^46,48,49^ All experimental signatures of Type B shifts were shown to depend on the length *L*_SC_, consistent with a flux quantum threading an area *L*_SC_*d* underneath the superconducting leads. The experimentally obtained value of *d* = 15 nm agrees with the separation between the Al and InAs layers (13.4 nm), up to some flux penetration into each layer. We therefore conclude that orbital effects strongly contributed to inducing Type B phase shifts. Type B shifts were observed for in-plane fields *B*_∥_ < 1 T, much lower than the values *B*_∥_ ≳ 9 T expected for InAs/Al heterostructures.^34^ We explain this by orbital effects, which were responsible for the induced gap reduction, forcing ABSs to move closer in energy. When the induced gap was most strongly suppressed, ABSs could cross, even with small Zeeman splitting. Type B shifts are expected to have only a weak dependence on the top-gate voltage, since *B*_∥_^Φ^ depends on the length of the superconducting leads and not on the properties of the junction region. Previous work reported similar phase shifts,^34^ where a π jump in the junction phase was accompanied by a minimum in the switching current. However, phase shifts depended on the top-gate voltage, unlike the Type B shifts reported here. This shows that orbital effects alone are not sufficient to explain the results of ref (34).

## Conclusions

In conclusion, measurements of the current–phase relation and Andreev bound state spectrum in hybrid quantum interference devices showed phase shifts with two distinct characters, termed Types A and B. Type A phase shifts are attributed to coupling of the external magnetic field with an internal Rashba spin–orbit field, resulting in a φ_0_-junction. Highly transmissive bound states were shown to make a significant contribution to the phase shift, which was much larger than expected for a single ballistic channel. The discrepancy might be due to the presence of many transverse modes, which future studies could investigate by varying the junction dimensions or by isolating individual modes using gate voltages.^64^ Type B shifts were consistent with a 0−π transition, where orbital effects in the superconducting leads played a critical role. This suggests that the geometry of the superconducting leads, and their impact on orbital effects, is a key ingredient for realizing π-junctions for superconducting electronics^65,66^ or in interpreting signatures of topological superconductivity.^30^ In particular, we show that orbital effects are crucial to explaining a minimum in the supercurrent, accompanied by the closure and reopening of the induced gap and a jump in the phase difference. Therefore, these signatures alone do not provide sufficient evidence for the realization of topological superconductivity.

## Methods

Devices were fabricated from a hybrid superconducting–semiconducting heterostructure grown by molecular beam epitaxy on a semi-insulating InP (001) substrate. The heterostructure consisted of a step-graded InAlAs buffer, onto which an In_0.75_Ga_0.25_As/InAs/In_0.75_Ga_0.25_As quantum well was grown with a termination of two GaAs monolayers. The step-graded metamorphic buffer compensated for the lattice mismatch between the InP and InAs, while the GaAs capping layers provided a barrier for In diffusion into the superconducting layer. The 8 nm InAs layer hosted a two-dimensional electron gas (2DEG), buried 13.4 nm below the semiconductor surface, as measured by transmission electron microscopy.^51^ A 15 nm layer of Al was deposited onto the semiconductor surface, *in situ* without breaking the vacuum in the growth chamber. Measurements of a gated Hall bar in this material showed a peak mobility of 18 000 cm^2^V^–1^s^–1^ at an electron sheet density of 8 × 10^11^ cm^–2^. This gave an electron mean free path of *l*_e_ ≳ 260 nm, implying that all Josephson junctions measured in this work were in the ballistic regime along the length *L* of the junction. We consider an in-plane Rashba spin–orbit field, as expected for electrons in [100] quantum wells in III–V systems.^55^ Since the Rashba field points in a perpendicular direction to the wavevector, we assume that it predominantly points in the direction perpendicular to the current flow.

The first step in patterning SQUIDs was to isolate each device from its neighbors by etching large mesa structures. This was done by selectively removing the Al layer with Transene type D, followed by a 380 nm chemical etch into the III–V heterostructure using a 220:55:3:3 solution of H_2_O:C_6_H_8_O_7_:H_3_PO_4_:H_2_O_2_. The second step was to pattern the Al device features by wet etching in Transene type D at 50 °C for 4 s. A dielectric layer of Al_2_O_3_ (3 nm) and HfO_2_ (15 nm) was deposited across the chip by atomic layer deposition; then gate electrodes were defined on top of the dielectric layer by evaporation and lift-off. Fine gate features were defined in a first step consisting of 5 nm Ti and 20 nm Au; a second deposition of Ti (10 nm) and Al (420 nm) connected the gates on top of the mesa structures to bonding pads, which were defined in the same step.

Measurements were performed in a dilution refrigerator with a base temperature at the mixing chamber below 10 mK. Magnetic fields were applied by using a three-axis vector magnet, nominally oriented perpendicular to the device (*B*_⊥_) and in the plane of the device (*B*_∥_, *B*_t_). Magnetic fields applied in the direction parallel to the Rashba spin–orbit field or equivalently the direction perpendicular to the current flow are denoted by *B*_∥_. The in-plane field was rotated by 90° to give *B*_t_, perpendicular to the spin–orbit field.

Measurements of the differential conductance were performed with standard lock-in amplifier techniques. An AC voltage *V*_AC_ = 3 μV was applied to the contact of the superconducting probe with a frequency of 311 Hz, in addition to a DC source–drain voltage *V*_SD_. The AC current *I*_1_ and DC current *I*_SD_ flowing through the probe to ground were measured via a current-to-voltage (*I*–*V*) converter. The differential voltage across the tunnel barrier *V*_1_ was measured to give a differential conductance *G* ≡ *I*_1_/*V*_1_. The transparency of the tunnel barrier was controlled with the gate voltages (*V*_T,L_, *V*_T,R_), which are denoted by *V*_T_ ≡ *V*_T,L_ = *V*_T,R_ (symmetric configuration). Measurements were performed in the tunneling regime, where *G* ≪ *G*_0_ = 2*e*^2^/*h*. A constant bias offset of 43 μV was subtracted from all data sets, due to a DC offset at the *I*–*V* converter. Since the tunnel probe was superconducting, the measured conductance was a convolution of the density of states (DoS) in the probe and SNS junction: *G* = DoS_Probe_ ⊗ DoS_SNS_. This amounted to a shift in the DoS_SNS_ features by ±*e*Δ*. For elevated in-plane magnetic fields, the superconducting gap in the tunnel probe was softened, leading to a finite DoS at low energy. This enabled measurements of the DoS in the SNS junction using an effectively normal probe, such that the measured conductance was directly proportional to the DoS in the SNS junction.^61,62^ In addition to conductance peaks at high source–drain bias corresponding to ABSs, we can attribute some features in the conductance spectrum to multiple Andreev reflections or to disorder in the tunnel barrier and sub-gap states in the DoS of the tunnel probe.^67^ For tunneling spectroscopy measurements at an in-plane magnetic field, a first calibration measurement was performed at each field value by sweeping the perpendicular field across a range > ±3 mT. The position of zero perpendicular field was determined from spectroscopic features, including the size of the superconducting gap, the shape and peak conductance of high-bias features, and the sharpness of spectral lines. Then, each spectroscopic map was taken across more than 5 oscillation periods such that spectral features were consistent over the full range.

Current-biased measurements were performed on the same device. Both contacts at the superconducting probe were floated such that no current flowed through the probe. The tunnel barrier gate voltages, which also covered large areas of the superconducting loop, were set to *V*_T_ = −1.5 V to deplete the InAs surrounding the Al features, thereby preventing parallel conduction and forming a well-defined current path. A DC current was applied by symmetrically biasing the SQUID loop such that the device potential was not raised with respect to the ground. Hence, the nominal voltage applied to the gate electrodes was the same as the potential difference between gates and the device. A ramped current signal was applied from a waveform generator at a frequency of 133 Hz. The voltage drop *V*_2_ across the loop was measured with an oscilloscope. The switching current, namely the current at which the SQUID transitioned from the superconducting to the resistive state, was recorded when *V*_2_ exceeded a voltage threshold of less than 15% of the maximum voltage in the resistive state. This measurement was repeated 32 times, and the resulting switching current values were averaged to account for stochastic fluctuations in the switching current.^60^ Values of the switching current reported in this work were averaged between values obtained for positive and negative bias currents *I*_DC_. Despite large bias currents (∼40 μA), the small normal state resistance (∼50 Ω) meant that small powers (<0.1 μW) were dissipated at the sample; no Joule heating effects were observed.

## Data Availability

The data presented in this study are available at https://zenodo.org/record/8298574. Further data that support the findings of this work are available from the corresponding author upon reasonable request.

## Supporting Information Available

Additional details on reference device measurements; extraction of the current phase relation and phase shift from switching current measurements; current–phase relation measurements in an in-plane magnetic field transverse to the junction axis, along *B*_t_; discussion of the origin of zero bias peaks in tunneling spectroscopy; additional tunneling spectroscopy measurements as a function of transverse in-plane field *B*_t_, at different top-gate voltages *V*_TG_ and in an additional device with large superconducting lead length *L*_SC_; additional measurements of the Type B phase shift in different devices; and a discussion of the kinetic inductance of the superconducting loop (PDF)

## Notes

An initial draft of this work was uploaded to the arXiv preprint server on June 5, 2023, as the following reference: Haxell, D. Z.; Coraiola, M.; Sabonis, D.; Hinderling, M.; ten Kate, S. C.; Cheah, E.; Krizek, F.; Schott, R.; Wegscheider, W.; Nichele, F. Zeeman and Orbital Driven Phase Transitions in Planar Josephson Junctions. *arXiv*. **2023**, 2306.01514.10.48550/arXiv.2306.01514 (accessed June 5, 2023).
