# Supplementary material for: Zeeman- and Orbital-Driven Phase Shifts in Planar Josephson Junctions
Source: ACS Nano. 2023 Sep 11;17(18):18139–47. doi: 10.1021/acsnano.3c04957 (PMC10540266; doi:10.1021/acsnano.3c04957)
Supplement: Supplementary file 1 — nn3c04957_si_001.pdf [file nn3c04957_si_001.pdf]

# Supporting Information: Zeeman- and Orbital-Driven Phase Shifts in Planar Josephson Junctions

Daniel Z. Haxell,[†] Marco Coraiola,[†] Deividas Sabonis,[†] Manuel Hinderling,[†] Sofieke C. ten Kate,[†] Erik Cheah,[‡] Filip Krizek,[†,‡,¶] Rüdiger Schott,[‡] Werner Wegscheider,[‡] and Fabrizio Nichele[*,†]

†*IBM Research Europe - Zurich, 8803 Rüschlikon, Switzerland*

‡*Solid State Laboratory, ETH Zürich, 8093 Zürich, Switzerland*

¶*Institute of Physics, Czech Academy of Sciences, 162 00 Prague, Czech Republic*

E-mail: fni@zurich.ibm.com

August 25, 2023

*Keywords:* Hybrid materials, superconductor-semiconductor, phase transitions, orbital effect, spin-orbit interaction, 2DEG, $\varphi$-junction

# Contents

**1 Reference Device** **2**

**2 Extracting the Current-Phase Relation** **6**

**3 Type B Phase Shifts of Current-Phase Relation** **9**

**4 Current-Phase Relation Dependence on $B_t$** **12**

**5 Zero-Bias Peak in Tunneling Spectroscopy** **15**

**6 Tunneling Spectroscopy as Function of $B_t$** **17**

**7 Tunneling Spectroscopy for different top-gate voltages** **19**

**8 Tunneling Spectroscopy in Device 5** **22**

**9 Devices with Varying Superconducting Lead Length** **24**

**10 Type A Phase Shifts in the Current Phase Relation** **28**

**11 Type A Phase Shifts in Tunneling Spectroscopy** **32**

**12 Phase Shifts due to Kinetic Inductance of the Superconducting Loop** **32**

**13 Data Availability** **34**

**References** **34**


# 1 Reference Device

External magnetic fields were applied using a three-axis vector magnet, nominally aligned in-plane and perpendicular to the surface of the chip. However, small misalignments of the external magnet with respect to the chip mean that large in-plane fields resulted in a perpendicular component, causing a flux through the superconducting loop. To account for this, a Reference Device was fabricated on the same chip, consisting of two Al constrictions in parallel [see Fig. 1(c) of the Main Text]. Constrictions were designed with a relatively high aspect ratio to clearly define the narrow region of the Al, both for improved control in fabrication and consistency in average switching currents. Several lengths were tested

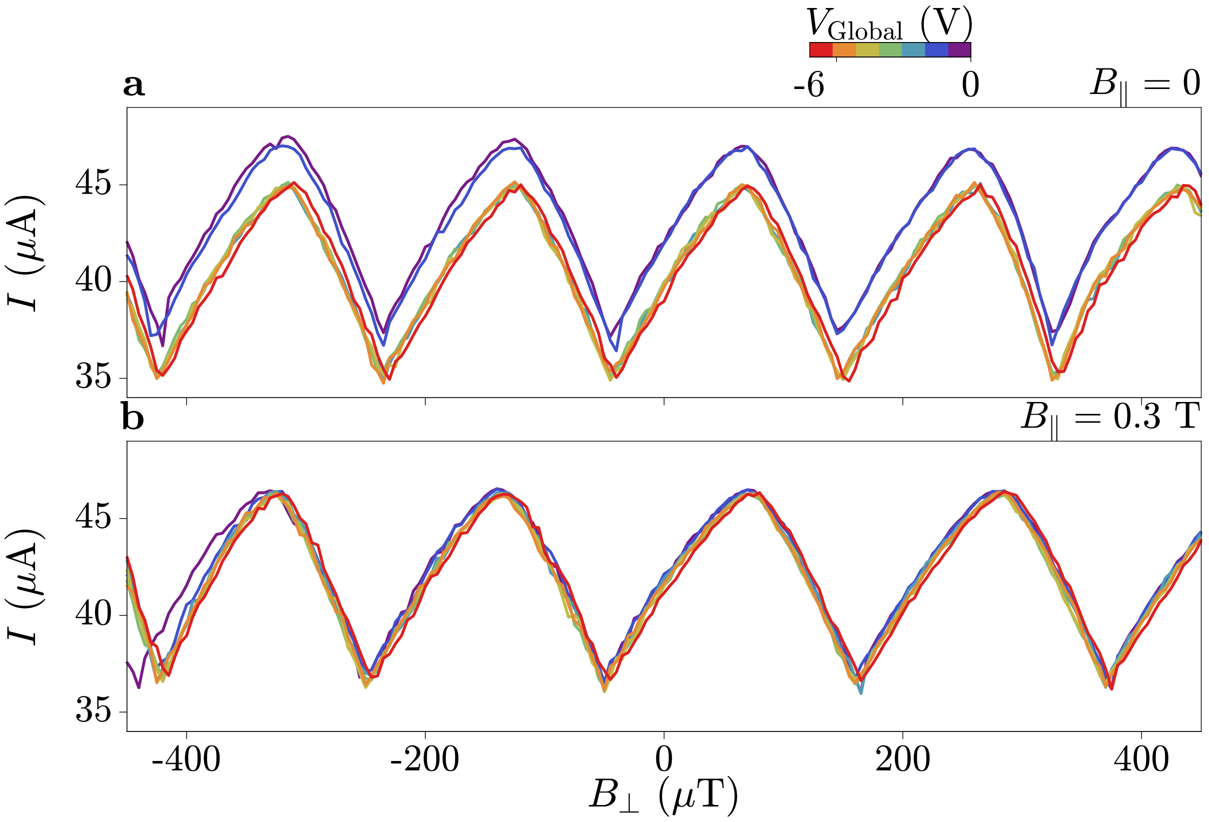


Figure S.1: (a) Switching current $I$ of the Reference Device as a function of perpendicular magnetic field $B_\perp$, for different values of global gate voltage $V_{\mathrm{Global}}$. Measurement performed at $B_\parallel = 0$. (b) Same as (a), at a finite in-plane magnetic field $B_\parallel = 0.3$ T.

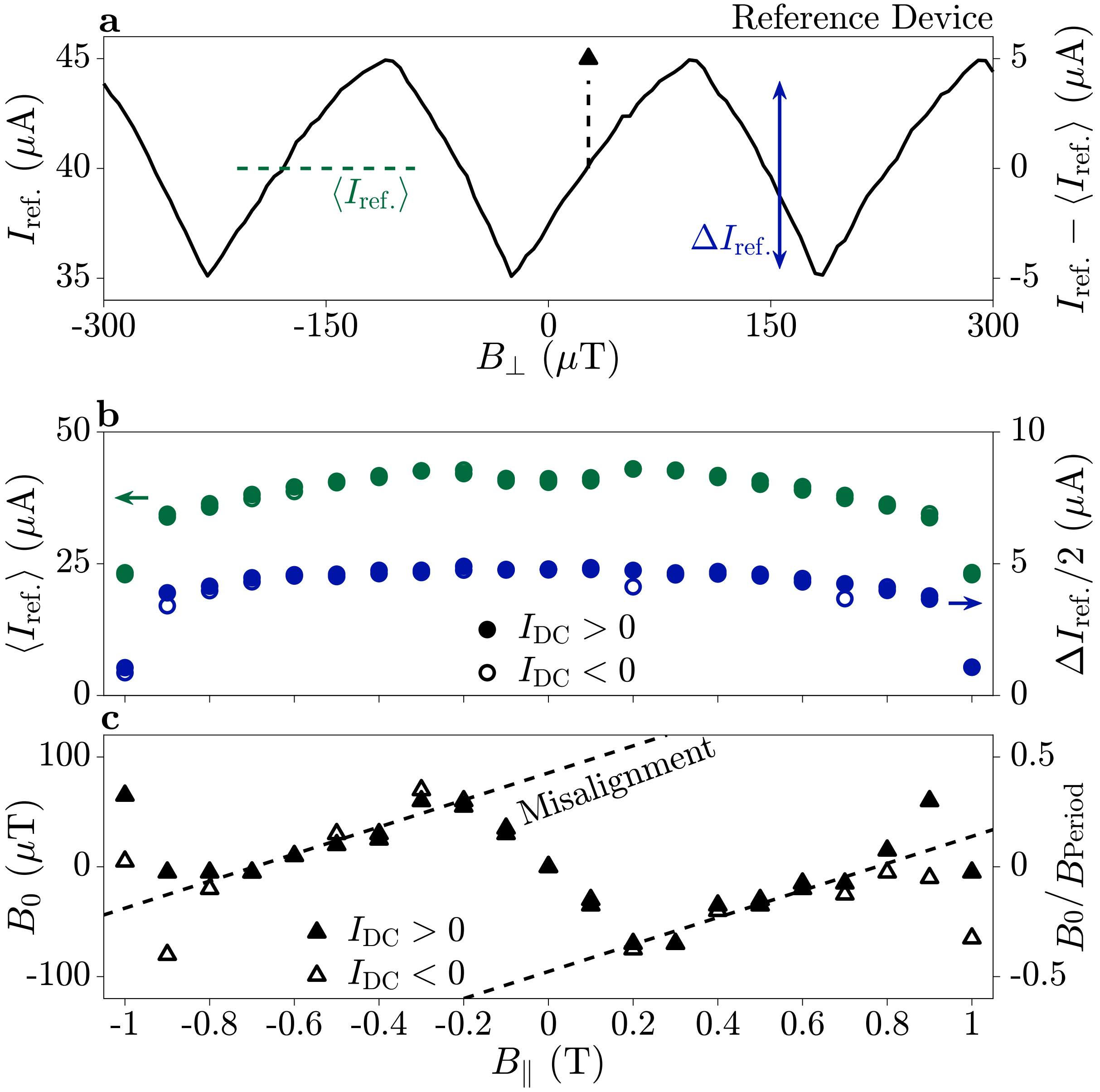


Figure S.2: (a) Current-phase relation of Reference Device $I_{\mathrm{ref.}}$ before (left) and after (right) subtraction of average $\langle I_{\mathrm{ref.}} \rangle$ (indicated by green dashed line). Amplitude of oscillations $\Delta I_{\mathrm{ref.}}$ is indicated by the blue arrow. Perpendicular field $B_\perp$ at which $I_{\mathrm{ref.}} - \langle I_{\mathrm{ref.}} \rangle = 0$, $B_0$, is indicated by the black triangle. (b) Average switching current of Reference Device $\langle I_{\mathrm{ref.}} \rangle$ (green, left axis) and half the oscillation amplitude $\Delta I_{\mathrm{ref.}}/2$ (blue, right axis) as a function of in-plane magnetic field $B_\parallel$. (c) Perpendicular field offset $B_0$ as a function of in-plane magnetic field $B_\parallel$ (left axis), and normalized to the oscillation period $B_{\mathrm{Period}}$ (right axis). Linear trend in $B_0$ for large $|B_\parallel|$ (dashed lines) are consistent with residual misalignment of the device chip with respect to the axis of the vector magnet, after appropriate calibration. Values in (b) and (c) are plotted for positive (negative) current bias $I_{\mathrm{DC}}$ as full (empty) markers.

in other devices, ranging from 200 nm to 1000 nm, with no appreciable change in device behavior. The Reference Device was covered with a global gate, to deplete the exposed areas on InAs surrounding the superconducting loop. Figure S.1 shows the switching current in the Reference Device as a function of perpendicular magnetic field, for different values of global gate voltage $V_{\mathrm{Global}}$ (colors). At zero in-plane magnetic field [Fig. S.1(a)], the average switching current and the amplitude of oscillations does not change for $V_{\mathrm{Global}} < -1.5$ V, which we associate with depletion of the InAs surrounding the device. Further, at $B_{\parallel} = 0.3$ T [Fig. S.1(b)] there was no shift in the oscillations as a function of $V_{\mathrm{Global}}$, consistent with the absence of spin-orbit effects in the Reference Device.

Measurements of the Reference Device were performed at $V_{\mathrm{Global}} = -3$ V, where the InAs surrounding the device was fully depleted. An example of the switching current of the Reference Device $I_{\mathrm{ref.}}$ is shown in Fig. S.2(a), as a function of perpendicular magnetic field $B_{\perp}$. The average switching current $\langle I_{\mathrm{ref.}} \rangle = 40$ $\mu$A (green dashed line) corresponds to the switching current of the wide Al constriction, $W_{\mathrm{cons.}} = 130$ nm, giving similar values to that of Device 1. The switching current after subtracting the average, $I_{\mathrm{ref.}} - \langle I_{\mathrm{ref.}} \rangle$ is shown on the right axis. The maximum switching current of the narrow Al constriction, $W_{\mathrm{cons.}} = 100$ nm, is inferred as half the peak-to-peak amplitude of oscillations, $\Delta I_{\mathrm{ref.}}/2$. The position where $I_{\mathrm{ref.}} - \langle I_{\mathrm{ref.}} \rangle = 0$ is assumed to be the perpendicular field at which there is no flux threading the loop, $B_0$ (marked by the triangle).

Figure S.2(b) shows the maximum switching current of the wide and narrow constriction as a function of in-plane magnetic field $B_{\parallel}$ (green and blue circles, respectively). Full (empty) markers correspond to the values obtained for positive (negative) applied current $I_{\mathrm{DC}}$. At $B_{\parallel} = 0$, the switching current appears to be slightly suppressed relative to that at a small in-plane field. This is attributed to a change in the interplay between quasiparticle populations in the superconductor and the number of quasiparticle relaxation channels in the superconducting leads.[1,2] At zero magnetic field, quasiparticles in the Al constriction are confined, with few relaxation channels in the superconducting leads, causing a suppression

in the superconducting gap. At small magnetic fields, quasiparticles are generated in the large superconducting leads connected to the constrictions, providing additional relaxation channels for quasiparticles in the constriction region. This partially alleviates the suppression of the superconducting gap relative to the zero-field case, leading to an increase in the switching current. At larger magnetic fields, more quasiparticles are generated in the superconductor resulting in suppression of the switching current. This effect was observed for both in-plane and perpendicular magnetic fields. For $B_{\parallel} > 0.9$ T, a large reduction was observed in the switching current of both constrictions, presumably caused by some portion of the superconducting loop becoming resistive. For this reason, no further studies were performed in this regime.

The perpendicular magnetic field offset $B_0$ of the Reference Device as a function of in-plane magnetic field $B_{\parallel}$ is shown in Fig. S.2(c). Misalignment between the vector magnet and the chip is evident at large in-plane magnetic fields, as indicated by the dashed lines. This was considered to be identical for the Reference Device and Device 1 since both are on the same chip. At small $B_{\parallel}$, external fields were distorted, presumably due to flux-focusing effect by the large Al leads.[3] Flux-focusing effects in the Reference Device for in-plane fields directed along the junction axis, $B_{\parallel}$, were consistent with those measured in all devices.

# 2 Extracting the Current-Phase Relation

An example of the switching current of Device 1 is shown in Fig. S.3(a) (circles), as a function of perpendicular magnetic field $B_{\perp}$. The switching current oscillated with a period $B_{\mathrm{Period}} = \Phi_0/A = 200\ \mu$T, where $\Phi_0 = h/2$e is the superconducting magnetic flux quantum and $A = 10.2\ (\mu\mathrm{m})^2$ is the area enclosed by the superconducting loop. The oscillation period of the Reference Device was always within 9 $\mu$T of $B_{\mathrm{Period}}$ in Device 1, corresponding to $< 5\%$ of the oscillation period. This is consistent with an almost identical area $A$ enclosed by the superconducting loop in the two devices.

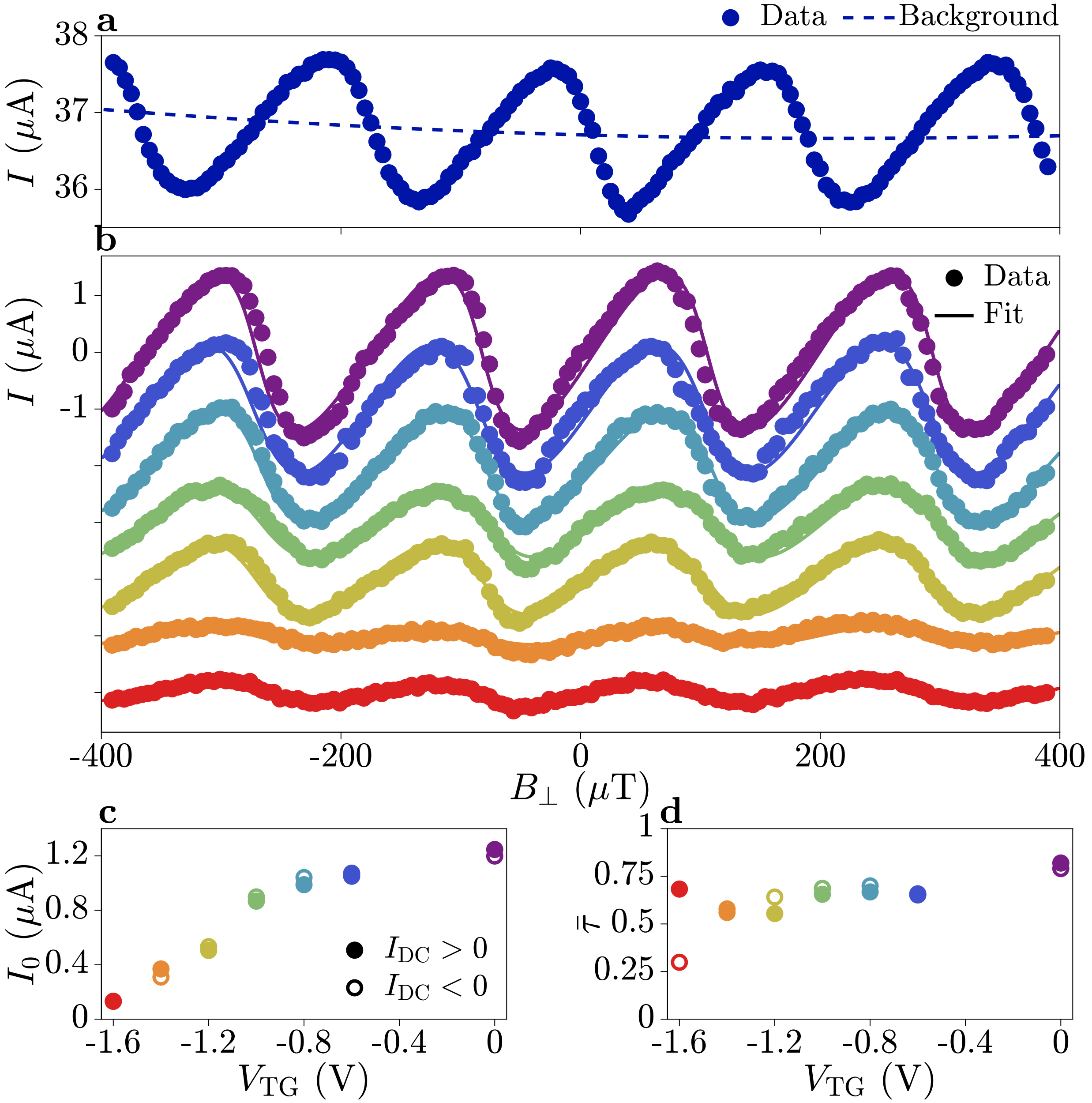


Figure S.3: (a) Switching current $I$ of Device 1 as a function of perpendicular magnetic field $B_\perp$. Data (circles) is fitted with a polynomial (dashed line) to extract the background switching current corresponding to the Al constriction. (b) Switching current after background extraction, as a function of perpendicular magnetic field $B_\perp$ for different top-gate voltages $V_{TG}$ [colors, defined in (c)]. Data (circles) is fitted with a formula for the current-phase relation of Andreev bound states (line). Each trace is offset by 1 $\mu$A. (c, d) Results of the fits presented in (b): maximum switching current $I_C$ and transmission $\tau$, for (c) and (d) respectively. Results for positive (negative) applied current $I_{DC}$ plotted as full (empty) markers.

A slowly-varying background is associated with the switching current of the Al constriction, which had a large switching current of $I \approx 37\ \mu$A. A weak dependence of the background switching current on $B_\perp$ is consistent with a change in the number and distribution of quasiparticle relaxation channels, as described in the previous section.[1,2] To remove this background, the data was fitted with a polynomial function over four complete periods. This background is shown as the dashed line in Fig. S.3(a). Due to the large asymmetry between the critical currents of the SNS junction and the Al constriction, the current-phase relation (CPR) of the SNS junction was taken to be the switching current of the SQUID after subtracting the background. This is plotted as the circles in Fig. S.3(b), at $B_\parallel = 0$ for different top-gate voltages $V_\mathrm{TG}$ [denoted by color, defined in Fig. S.3(c)]. The data showed a large forward skewness, consistent with the presence of highly transmissive ABSs in the junction.[4]

The CPR of an SNS junction containing $N$ modes is described by

$$I(\varphi) = -\frac{2\mathrm{e}}{\hbar}\sum_{n=1}^{N}\frac{\partial E_{\mathrm{A},n}(\varphi)}{\partial\varphi}, \tag{S.1}$$

where $E_{\mathrm{A},n} = \mathit{\Delta}\sqrt{1-\tau_n\sin^2(\varphi/2)}$ is the energy of the $n^\mathrm{th}$ ABS with transmission $\tau_n$, $\Delta$ is the superconducting gap and $\varphi$ is the phase difference across the SNS junction. The total supercurrent is a sum over the contributions of each ABS in the junction. The junctions studied in this work all had a large width $W = 2.5\ \mu$m, and therefore contained many transverse conducting modes. Since detailed knowledge about individual modes is missing, we instead consider an effective transmission $\bar{\tau}$ to describe the properties of the CPR: the transmission which would reproduce the CPR in a junction where all modes have identical transmission. With the application of an in-plane magnetic field, the CPR is expected to obtain a phase shift $\varphi_0$.[5] Accounting for these considerations, we obtain the equation

$$I(\varphi) = I_\mathrm{N}\frac{\bar{\tau}\sin(\varphi-\varphi_0)}{E_\mathrm{A}(\varphi-\varphi_0)/\Delta}, \tag{S.2}$$

where $I_{\mathrm{N}} = (\mathrm{e}/2\hbar)\bar{N}\Delta$ and $\bar{N}$ is the effective number of modes in the junction. The phase difference across the junction is related to the perpendicular magnetic field by $\varphi = 2\pi(B_{\perp} \cdot A/\Phi_0)$. The switching current as a function of perpendicular magnetic field is therefore fitted using Eq. S.2 obtaining three parameters: $I_0$, $\bar{\tau}$ and $\varphi_0 \equiv 2\pi(B_0 \cdot A/\Phi_0)$. The maximum switching current $I_0$ is not necessarily equal to $I_{\mathrm{N}}$, so it is obtained as the maximum of $I(\varphi)$ from the fit.

The fits to the data in Fig. S.3(b) are shown as the solid lines, with the maximum switching current $I_0$ and effective transmission $\bar{\tau}$ plotted in Figs. S.3(c) and (d), respectively. Note that $I_0$ is not necessarily equal to the critical current of the SNS junction, since stochastic fluctuations of the phase result in a switching current much lower than the critical current in planar Josephson junctions.[6] The maximum switching current decreased as a function of top-gate voltage $V_{\mathrm{TG}}$, until no oscillations were visible at $V_{\mathrm{TG}} < -1.6$ V. The effective transmission did not change appreciably across this range, indicating the presence of highly transmissive ABSs across the full gate range. Results are plotted for positive ($I_{\mathrm{DC}} > 0$) and negative ($I_{\mathrm{DC}} < 0$) bias current directions, as the full and empty markers respectively. Changing the current direction resulted in a reversal of the skewness of the CPR, since the external perpendicular field $B_{\perp}$ had a fixed direction. The sign of the phase $\varphi$ used in Eq. S.2 was therefore reversed for negative $I_{\mathrm{DC}}$, as was the associated value of $B_0$ coming from the fit. This meant that a larger $\varphi_0$ always corresponded to a larger $B_0$, independent of the current direction.

# 3 Type B Phase Shifts of Current-Phase Relation

At a given in-plane magnetic field $B_{\parallel}$, the CPR of the SQUID was found by measuring the switching current as a function of perpendicular field $B_{\perp}$, which was swept multiple times across a small range such that it was stable. The switching current was measured for positive and negative currents, before changing the top-gate voltage $V_{\mathrm{TG}}$. Once the switching current

had been collected for all top-gate voltages, $B_{\|}$ was ramped to the next value. The in-plane field was always swept away from $B_{\|} = 0$, such that sweeps in the positive and negative $B_{\|}$ directions began at $B_{\|} = 0$. As such, all measurements are relative to the values obtained at zero in-plane field in that field sweep. Since fitting with Eq. S.2 always returned values for $\varphi_0$ in the range $[-\pi,\ \pi]$, results at a given in-plane field were shifted by integer multiples of the oscillation period $B_{\mathrm{Period}}$ such that $B_0$ values followed a monotonic trend. The magnetic field $B_{\|}$ was swept multiple times, from $-1$ T to 1 T, before measurements were taken to minimize hysteresis effects. Nevertheless, some hysteresis was observed at $B_{\|} = 0$, where flux focusing effects were most prevalent. Hence, results for $B_{\|} > 0$ and $B_{\|} < 0$ were combined such that current-averaged $B_0$ features were symmetric for $|B_{\|}| \geq 0.1$ T. Since all measurements were plotted with respect to the value at $B_{\|} = 0$, the trend in $B_0$ is compared between each device and the Reference Device. In this way, shifts in the trend of $B_0$ can be reliably compared in each device. The results of Figs. 1 and 3 of the Main Text were plotted following this procedure. An identical procedure was followed for in-plane magnetic fields applied transverse to the junction axis, $B_{\mathrm{t}}$.

The CPR as a function of in-plane magnetic field $B_{\|}$ is plotted in Fig. S.4, where each CPR is normalized to the maximum switching current $I_0$ at that value of $B_{\|}$. The top-gate voltage was $V_{\mathrm{TG}} = -1$ V, the same as in the tunneling spectroscopy maps of Fig. 3 in the Main Text. Each CPR trace is plotted with respect to $B_0$ of the Reference Device at that in-plane field [see Fig. S.2(c)], indicated by the vertical dashed line at $B_{\perp} = 0$. The position of zero current through the SNS junction is marked by the second dashed line, which encloses the shaded green area to $B_{\perp} = 0$. The phase shift shown in Fig. 1(f) of the Main Text is evident, increasing to $\Delta B_0/B_{\mathrm{Period}} \approx 0.5$ at $B_{\|} = 0.6$ T, where the switching current is minimal [see Fig. 1(e) of the Main Text]. For larger $B_{\|}$, the phase offset moves towards zero, or equivalently towards $\Delta B_0/B_{\mathrm{Period}} = 1$ as shown in Fig. 1(f) of the Main Text.

This result is consistent with the interpretation of a large phase shift induced by orbital effects in the superconducting leads. As the superconducting gap in the leads is suppressed

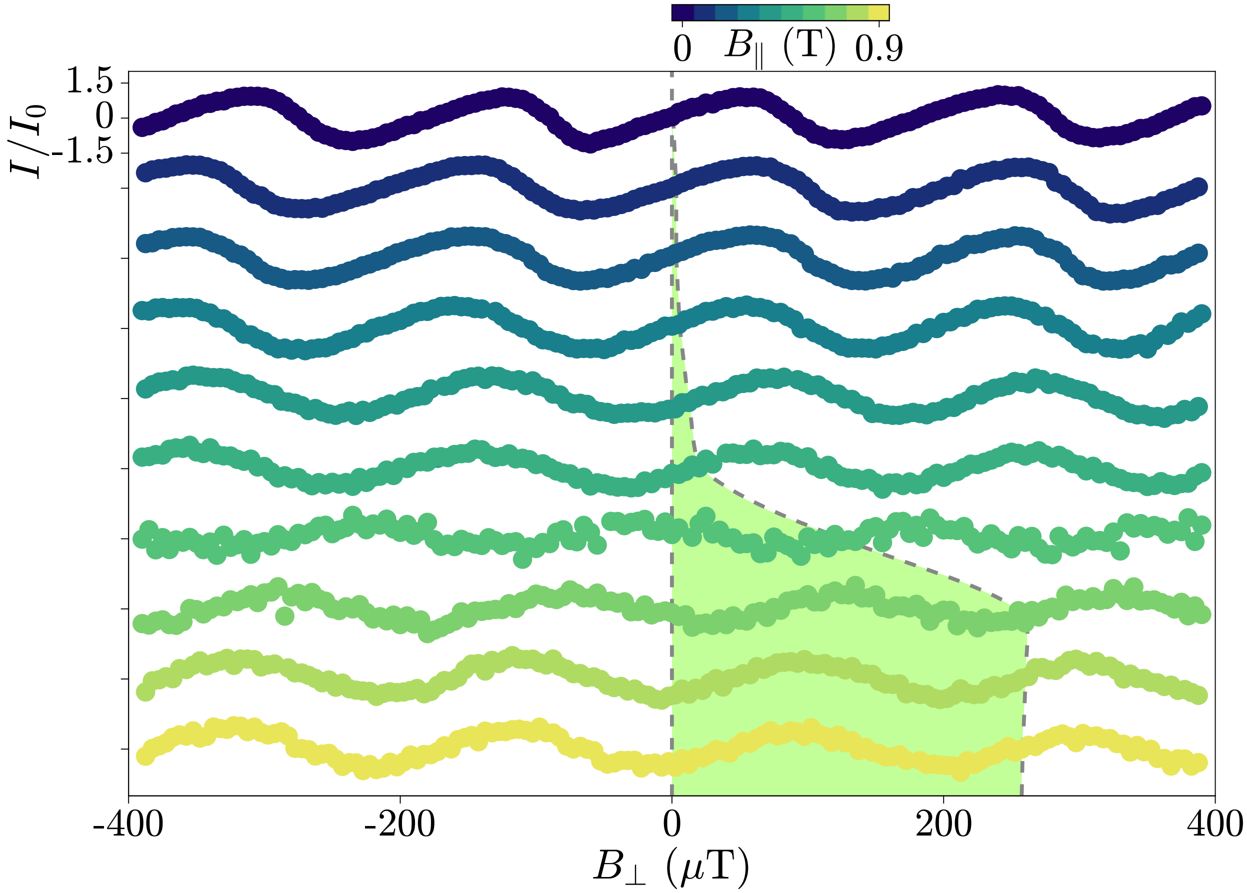


Figure S.4: Current-phase relation (CPR) for increasing in-plane magnetic field $B_{\parallel}$. Currents $I$ are normalized to the maximum switching current $I_0$ at each $B_{\parallel}$. CPR traces are offset by the perpendicular field offset $B_0$ of the Reference Device at the corresponding in-plane field $B_{\parallel}$. The shift in $B_{\perp}$ of the zero-current position is indicated by the green shading, between the two grey dashed lines. Datapoints where the switching current was significantly lower than its neighbors were removed, since they correspond to early switching events in the device by stochastic fluctuations.[6] Each trace is offset by 3 $\mu$A to improve visibility.

by orbital effects, ABSs in the junction are pushed closer together, such that some cross zero energy due to Zeeman splitting at the finite in-plane field. When the superconducting gap is sufficiently small, most states have sufficient energy splitting that the ground state is at $\varphi = \pi$ rather than $\varphi = 0$.[5] This explains the phase shift of $\varphi = 2\pi(\Delta B_0/B_{\text{Period}}) \approx \pi$ at $B_{\|} = 0.6$ T, where the orbital effects are strongest. For $B_{\|} > 0.6$ T, the superconducting gap in the leads increases as the orbital effects become weaker. This means that fewer ABSs have sufficient energy splitting to shift the phase of the ground state, and $\varphi_0$ moves away from $\pi$.

Neither the magnitude of Type B shifts, nor the magnetic field at which they are most pronounced, depended on top-gate voltage. This is consistent with orbital effects as the dominant cause of phase shifts, since the field at which one flux quantum threads the area underneath the superconducting leads depends only on the lead size and not on the junction properties. Although the number and transmission of ABSs changes as a function of top-gate voltage, sub-gap states only cross when the induced gap is largely suppressed by orbital effects. Nevertheless, the observed phase shift extends over a range of in-plane fields. This might be attributed to the many ABSs in the junction, each with a different transmission, which therefore cross at different Zeeman energies. By changing the number of ABSs with a gate voltage, the exact dependence of the Type B shift might change via this effect. There might be indications of this trend in Fig. 1(f) of the Main Text. However, the most clear feature is that the switching current has a minimum at $B_{\|}^{\Phi} = 0.6$ T, with a corresponding phase shift of $\varphi_0 \approx \pm\pi$, independent of top-gate voltage.

# 4 Current-Phase Relation Dependence on $B_{\text{t}}$

Phase shifts induced by orbital effects rely on an in-plane magnetic field generating a flux underneath the superconducting leads. This is particular for in-plane fields applied along the junction axis ($B_{\|}$), since a field applied in a perpendicular direction ($B_{\text{t}}$) would not generate

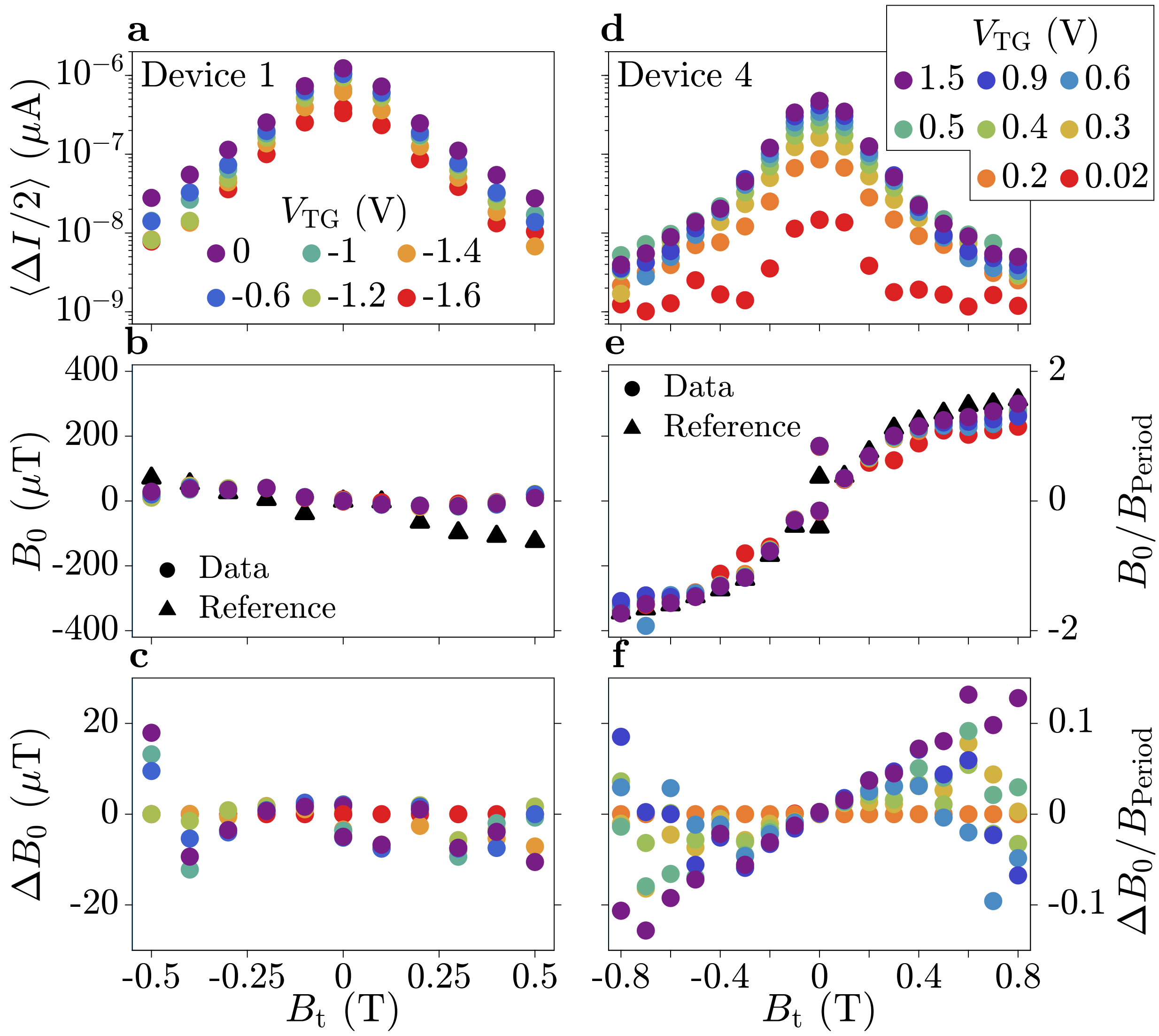


Figure S.5: (a) Half-amplitude of switching current oscillations, $\langle \Delta I/2 \rangle$, for different top-gate voltages $V_{\mathrm{TG}}$, as a function of in-plane magnetic field $B_{\mathrm{t}}$. No oscillations in switching current were observed for $|B_{\mathrm{t}}| > 0.5$ T. (b) Perpendicular field offset $B_0$ of switching current oscillations as a function of $B_{\mathrm{t}}$. The field offset normalized to the oscillation period, $B_0/B_{\mathrm{Period}}$, is plotted on the right axis. Datapoints for Device 1 (Reference Device) correspond to circles (triangles). The $B_0$ for Device 1 and the Reference Device do not align, due to the different level of flux focusing in the two devices. (c) Perpendicular field offset $B_0$ plotted with respect to that for the most negative top-gate voltage, $V_{\mathrm{TG}} = -1.6$ V. There is no discernable gate-dependent shift across the measured range. (d) Switching current of Device 4 as a function of in-plane magnetic field $B_{\mathrm{t}}$, for different top-gate voltages $V_{\mathrm{TG}}$. No $B_{\perp}$-dependent oscillations in switching current were observed for $|B_{\mathrm{t}}| > 0.8$ T. (e) Perpendicular magnetic field offset $B_0$ in Device 4 (circles) and the associated Reference Device (triangles), as a function of $B_{\mathrm{t}}$. (f) Field offset $B_0$ relative to that at $V_{\mathrm{TG}} = 0.2$ V, as a function of $B_{\mathrm{t}}$. Comparison made to $V_{\mathrm{TG}} = 0.2$ V rather than the most negative, $V_{\mathrm{TG}} = 0.02$ V, due to the comparatively large deviation of this datapoint from the Reference Device.

destructive interference of ABSs in the superconducting leads.[7] A strong direction dependence is also predicted for spin-orbit related effects. An in-plane Rashba field is expected for this InAs heterostructure,[8] due to the strong electric field component perpendicular to the quantum well. This gives a Hamiltonian of the form $H_{\rm SO} = \alpha(k_y\sigma_x - k_x\sigma_y)$, where $(k_x, k_y)$ are the in-plane wavevector components, $(\sigma_x, \sigma_y)$ are the in-plane spin components and $\alpha$ is the spin-orbit coupling strength.[9] We expect the Rashba field to point in a direction perpendicular to the wavevector, and therefore perpendicular to the direction of current flow. This corresponds to the $B_{\|}$ direction of the in-plane magnetic field. This direction-dependence has implications for anomalous phase shifts, as well as proposed topological transitions where angular dependence is a crucial ingredient.[10]

Figure S.5(a) shows the maximum switching current of SQUID oscillations in Device 1 as a function of in-plane field $B_{\rm t}$. The maximum switching current decreased for larger $|B_{\rm t}|$, until no oscillations in the switching current were observed for $|B_{\rm t}| > 0.5$ T. No minimum and increase in the switching current was observed, nor was there any associated phase jump [Fig. S.5(b)], unlike for $B_{\|}$ [see Figs. 1(e, f) of the Main Text]. This is consistent with a lack of orbital effects in the superconducting leads. The small difference between $B_0$ for Device 1 and the Reference Device, measured for the same applied $B_{\rm t}$, is attributed to different flux focusing effects between the two devices. Figure S.5(c) shows the perpendicular field offset relative to the most negative top-gate voltage, $V_{\rm TG} = -1.6$ V. No gate-dependence was present in $\Delta B_0$, and there was no linear trend as a function of in-plane field $B_{\rm t}$. The absence of gate-dependent phase shifts as a function of $B_{\rm t}$ supports the interpretation that Type B phase shifts for $B_{\|}$ are enabled by the presence of spin-orbit coupling.

Switching current measurements as a function of $B_{\rm t}$ were also performed on Device 4. Figure S.5(d) shows the maximum switching current as a function of $B_{\rm t}$, for different top-gate voltages $V_{\rm TG}$. No minimum and increase in the switching current was observed up to $B_{\rm t} = 0.8$ T, beyond which no oscillations in switching current were visible. The corresponding offset in perpendicular field $B_0$ [circles, Fig. S.5(e)] showed no deviation from that of the

Reference Device [triangles, Fig. S.5(e)]. This is consistent with Device 1 [Fig. S.5], supporting the conclusion that orbital effects do not play a role in measurements in in-plane fields applied perpendicular to the junction axis. Figure S.5(f) shows the perpendicular field offset relative to $V_{\mathrm{TG}} = 0.2$ V. This was chosen to be the reference in this case due to the large deviation of the $V_{\mathrm{TG}} = 0.02$ V data from the Reference Device. This was potentially due to the small switching currents at the lowest top-gate voltage, causing an unreliable fit result. Some gate-dependent trend is apparent in Fig. S.5(f), although with a smaller gradient than observed for $B_{\parallel}$ [see Fig. 1(f) of the Main Text]. This could be due to stray in-plane fields coupling to the primary spin-orbit direction, or to an additional spin-orbit component in the junction.

# 5 Zero-Bias Peak in Tunneling Spectroscopy

Tunneling spectroscopy measurements at large in-plane fields $B_{\parallel} \approx 0.8$ T show a peak in the differential conductance $G$ close to zero source-drain bias $V_{\mathrm{SD}}$ [see Fig. 3(e) of the Main Text]. In measurements of similar devices, a zero-bias peak (ZBP) has been associated with the emergence of a topological phase.[11,12] Here, we show additional data of the ZBP observed in Fig. 3(e) of the Main Text and comment on its origin.

Figures S.6(a-g) show the conductance $G$ as a function of perpendicular magnetic field $B_{\perp}$, for in-plane magnetic fields $B_{\parallel} > 0.6$ T (i.e., after the closure of the superconducting gap at $B_{\parallel} = 0.6$ T). Conductance maps show periodic lobe-like features: each map is plotted such that the center of a lobe is aligned to $B_{\perp} = 0$. The top-gate voltage was set to $V_{\mathrm{TG}} = -1$ V, identical to that in Fig. 3 of the Main Text [such that Fig. S.6(d) is the same as Fig. 3(e) of the Main Text]. A high-conductance feature is visible close to $V_{\mathrm{SD}} = 0$ in many maps, but does not appear robustly for all in-plane fields and is rarely well separated from conductance features at higher source-drain bias. To test the robustness of this ZBP, the magnetic field was fixed to $B_{\parallel} = 0.8$ T and $B_{\perp} = 0$, then the top-gate was varied from $V_{\mathrm{TG}} = -0.92$ V to

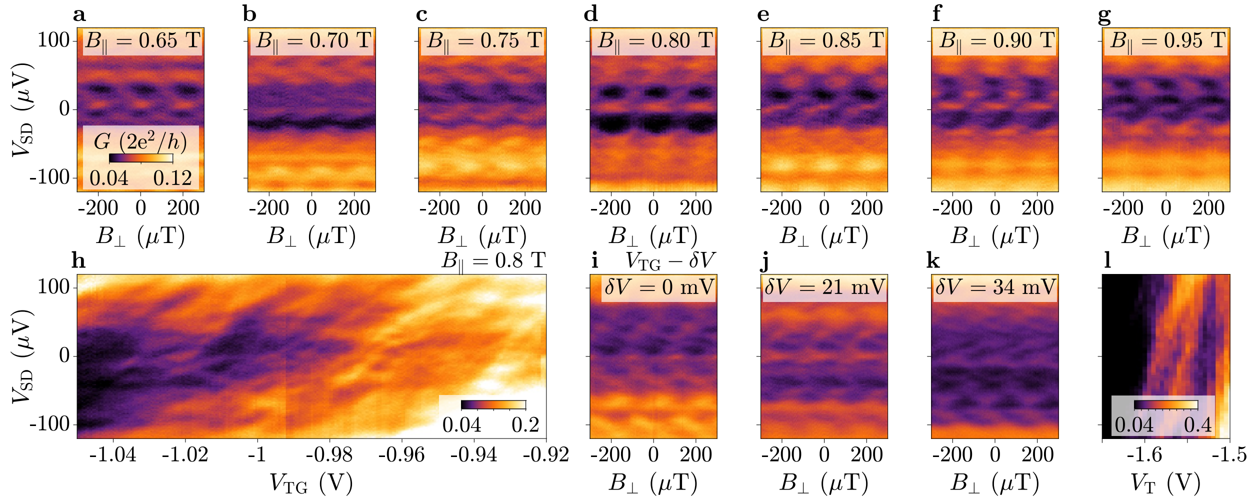


Figure S.6: (a-g) Differential conductance $G$ as a function of source-drain bias $V_{SD}$ and perpendicular magnetic field $B_\perp$, for different in-plane magnetic fields $B_\parallel$. The gate configuration was identical to that of Fig. 3 of the Main Text, with $V_{TG} = -1$ V [data plotted in (d) is identical to Fig. 3(e) of the Main Text]. (h) Differential conductance as a function of top-gate voltage $V_{TG}$, at $B_\parallel = 0.8$ T and $B_\perp = 0$. (i, k) Conductance maps as a function of perpendicular field $B_\perp$, at $B_\parallel = 0.8$ T. The top-gate voltage was set to $V_{TG} - \delta V$, where $V_{TG} = -1$ V and $\delta V = 0$, 21 and 34 mV for (i, k) respectively. (l) Differential conductance as a function of bias $V_{SD}$ and tunnel-gate voltage $V_T$, at $B_\parallel = 0.8$ T and $B_\perp = 0$. The top-gate voltage was set to $V_{TG} = -1$ V. High conductance features are tuned by $V_T$ across the full bias range.

$V_{\mathrm{TG}} = -1.05$ V [Fig. S.6(h)]. Conductance features moved close to $V_{\mathrm{SD}} = 0$ as a function of $V_{\mathrm{TG}}$, but were not stable at $V_{\mathrm{SD}} = 0$ for more than a few millivolts. Figures. S.6(i-k) show the differential conductance as a function of perpendicular field $B_{\perp}$, at top-gate voltages offset from $V_{\mathrm{TG}} = -1$ V by $-\delta V$, where $\delta V = 0$, 21 mV and 34 mV for (i-k) respectively. The conductance spectrum changed appreciably, and a high-conductance feature is evident in Fig. S.6(j) but not in the others. Note also that the regime of Fig. S.6(d) was not recovered in (i), despite the identical gate and field configuration. Figure S.6(l) shows the differential conductance $G$ as a function of tunnel-barrier gate voltage, $V_{\mathrm{T}}$. High-conductance features were dependent on $V_{\mathrm{T}}$, and moved across the low-bias region.

Zero-bias peaks were shown to be sensitive to in-plane mangetic fields $B_{\parallel}$ and top-gate voltage $V_{\mathrm{TG}}$, and tunnel-barrier-dependent conductance features were shown to move close to $V_{\mathrm{SD}} = 0$. These results suggest that ZBPs were most likely due to ABSs coalescing close to zero energy, rather than being topological in origin. This is despite the gap closure and opening, shown in Fig. 3 of the Main Text and associated with orbital effects in the superconducting leads. This result suggests that additional levels of caution are needed in interpreting ZBPs as indicative of a topological transition, even in the presence of gap closure and reopening. We note that the top-gate voltage $V_{\mathrm{TG}} = -1$ V was chosen to have good visibility of conductance features at low $B_{\parallel}$, to be in a regime of single-subband occupation (based on supercurrent measurements) and to match a value used in supercurrent measurements [see Fig. 1(e-h) in the Main Text]. It was not chosen based on the observation of a ZBP; the emergence of a ZBP after gap closure and reopening was by coincidence rather than by fine-tuning of $V_{\mathrm{TG}}$.

# 6 Tunneling Spectroscopy as Function of $B_{\mathrm{t}}$

Current-biased measurements for in-plane magnetic fields aligned perpendicular to the junction axis ($B_{\mathrm{t}}$) are supported by tunneling spectroscopy [see Fig. S.7]. Measurements were

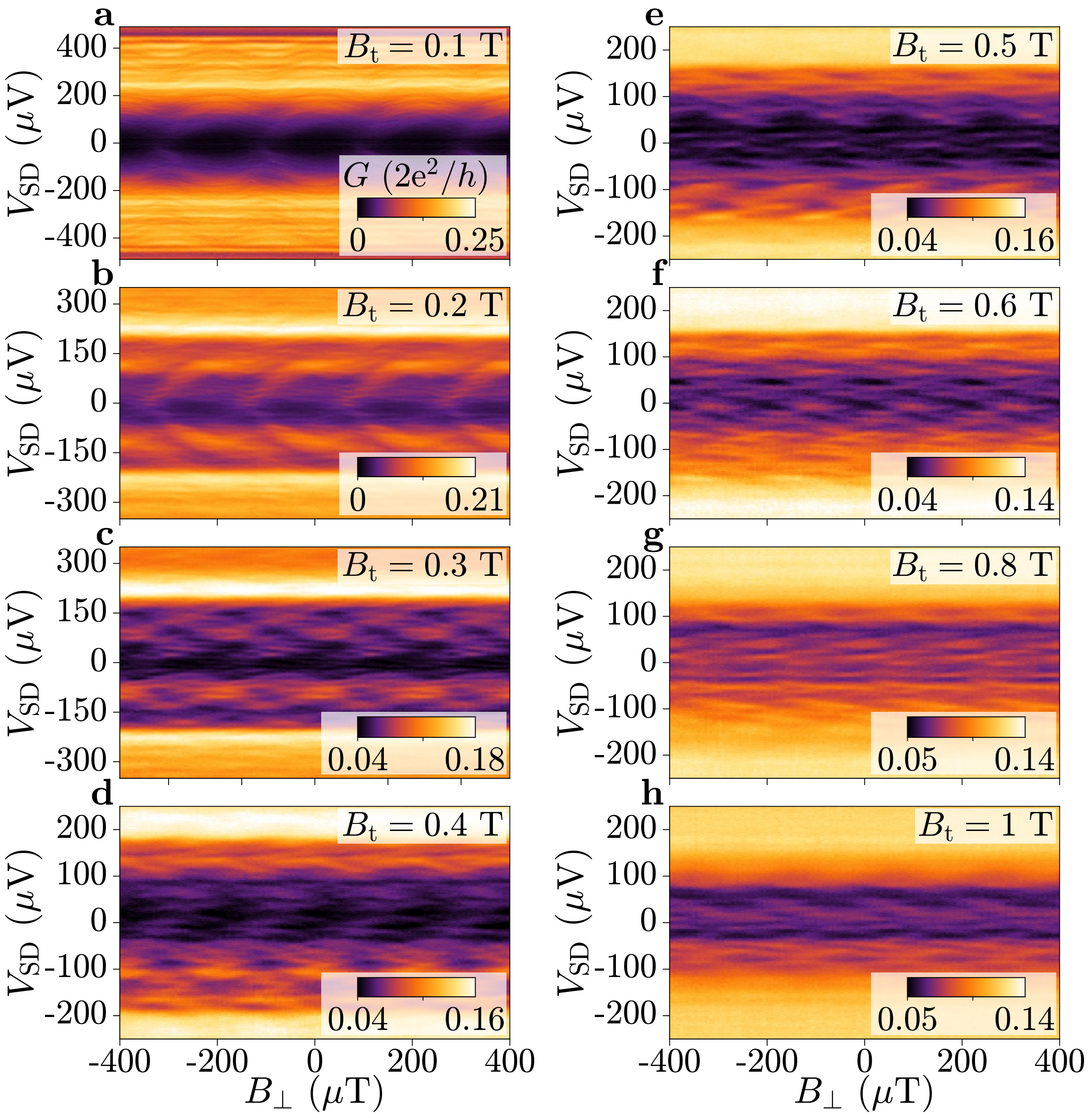


Figure S.7: Differential conductance $G$ as a function of source-drain bias voltage $V_{\mathrm{SD}}$ and perpendicular magnetic field $B_\perp$, for different values of in-plane mangetic field $B_{\mathrm{t}}$. Measurements were taken at $V_{\mathrm{TG}} = -1$ V, in an identical gate configuration as that of Fig. 3 of the Main Text.

taken with an identical gate voltage configuration to those in Fig. 3 of the Main Text. For small values of $B_\mathrm{t}$, superconductivity in the tunnel probe was quickly softened such that conductance features occurred at low bias $V_\mathrm{SD}$ [Figs. S.7(a, b)]. Conductance features were periodic with perpendicular magnetic field $B_\perp$, but with a weak dependence consistent with the small switching currents oberved in Fig. S.5. Conductance features did not resemble those of ABSs described by $E_\mathrm{A} = \Delta\sqrt{1 - \tau \sin^2(\varphi/2)}$ , instead forming a complex network and crossing $V_\mathrm{SD} = 0$ in many places [Figs. S.7(c, d)]. This became more pronounced at larger $B_\mathrm{t}$ [Figs. S.7(e, f)] until the superconducting gap was largely suppressed and conductance features changed very little with $B_\perp$ [Figs. S.7(g, h)]. No reopening of the superconducting gap was observed in these spectroscopic maps, up to large in-plane fields well beyond the value at which no oscillations in the switching current were visible. Conductance features are not well described by a simple model of ballistic ABSs in a short junction, instead showing crossings and interactions at high and low bias. These results indicate the absence of a phase transition, since there was no reopening of the superconducting gap. This is consistent with the lack of orbital effects for in-plane fields applied perpendicular to the junction axis. More sophisticated modeling of ABSs would be required to understand the conductance features in detail, which is beyond the scope of this work.

# 7 Tunneling Spectroscopy for different top-gate voltages

Figures S.8 and S.9 show tunneling spectroscopy maps for increasing in-plane magnetic field $B_\parallel$, at top-gate voltages of $V_\mathrm{TG} = -0.6$ V and $V_\mathrm{TG} = -1.4$ V respectively. The tunnel barrier gates were adjusted to be in the tunneling regime, so were set to $V_\mathrm{T} = -2.46$ V and $(V_\mathrm{T,L}, V_\mathrm{T,R}) = (-1.835, -1.805)$ V for Figs. S.8 and S.9 respectively. At $V_\mathrm{TG} = -0.6$ V, many more conductance features were present relative to $V_\mathrm{TG} = -1$ V [Fig. S.8(a) compared with Fig. 3(a) of the Main Text], consistent with more modes present in the junction. In

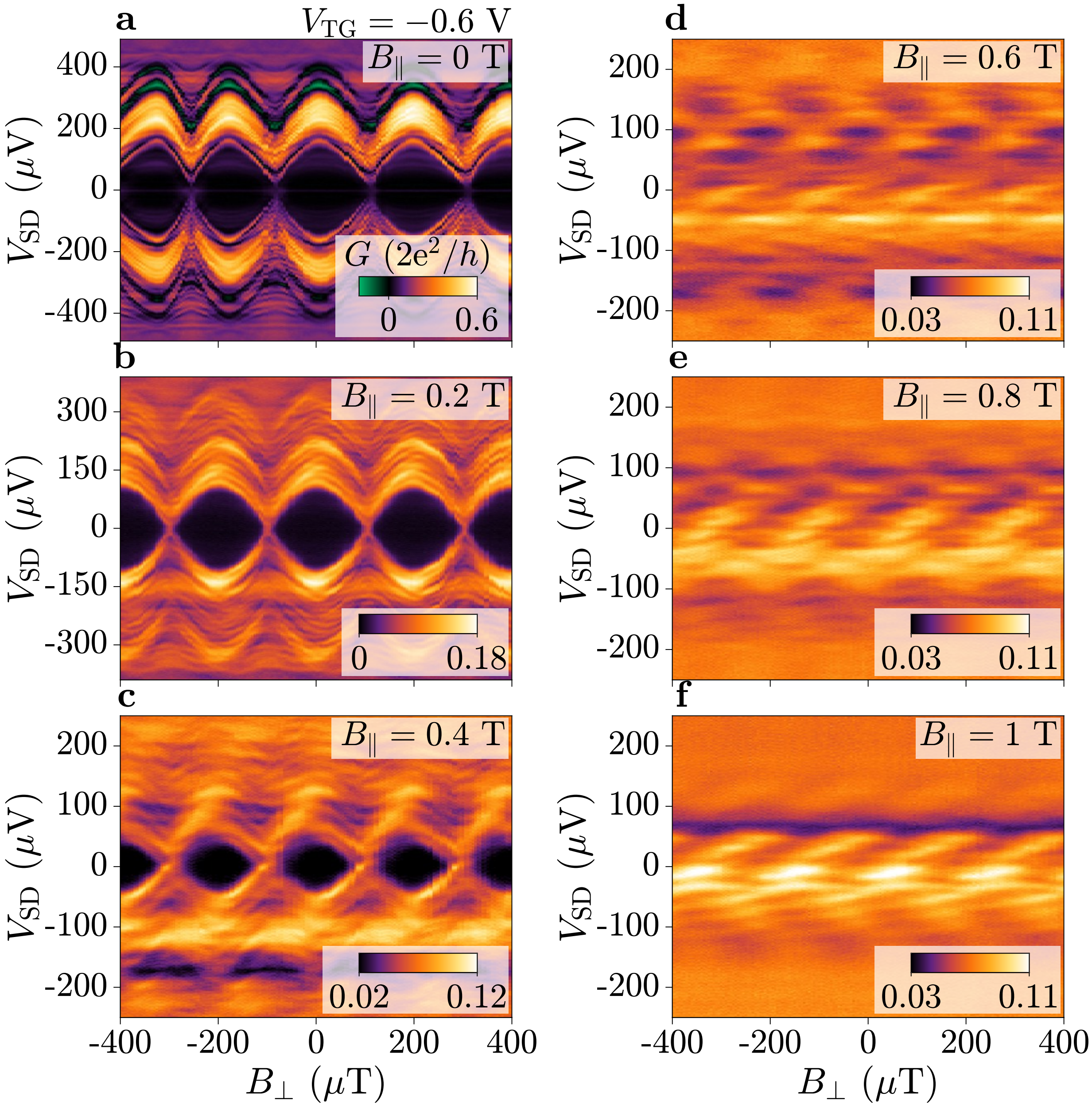


Figure S.8: Differential conductance $G$ as a function of source-drain bias $V_{SD}$ and perpendicular magnetic field $B_\perp$, for different values of in-plane magnetic field $B_\parallel$. Taken at a top-gate voltage of $V_{TG} = -0.6$ V and tunnel-gate voltage $V_T = -2.46$ V.

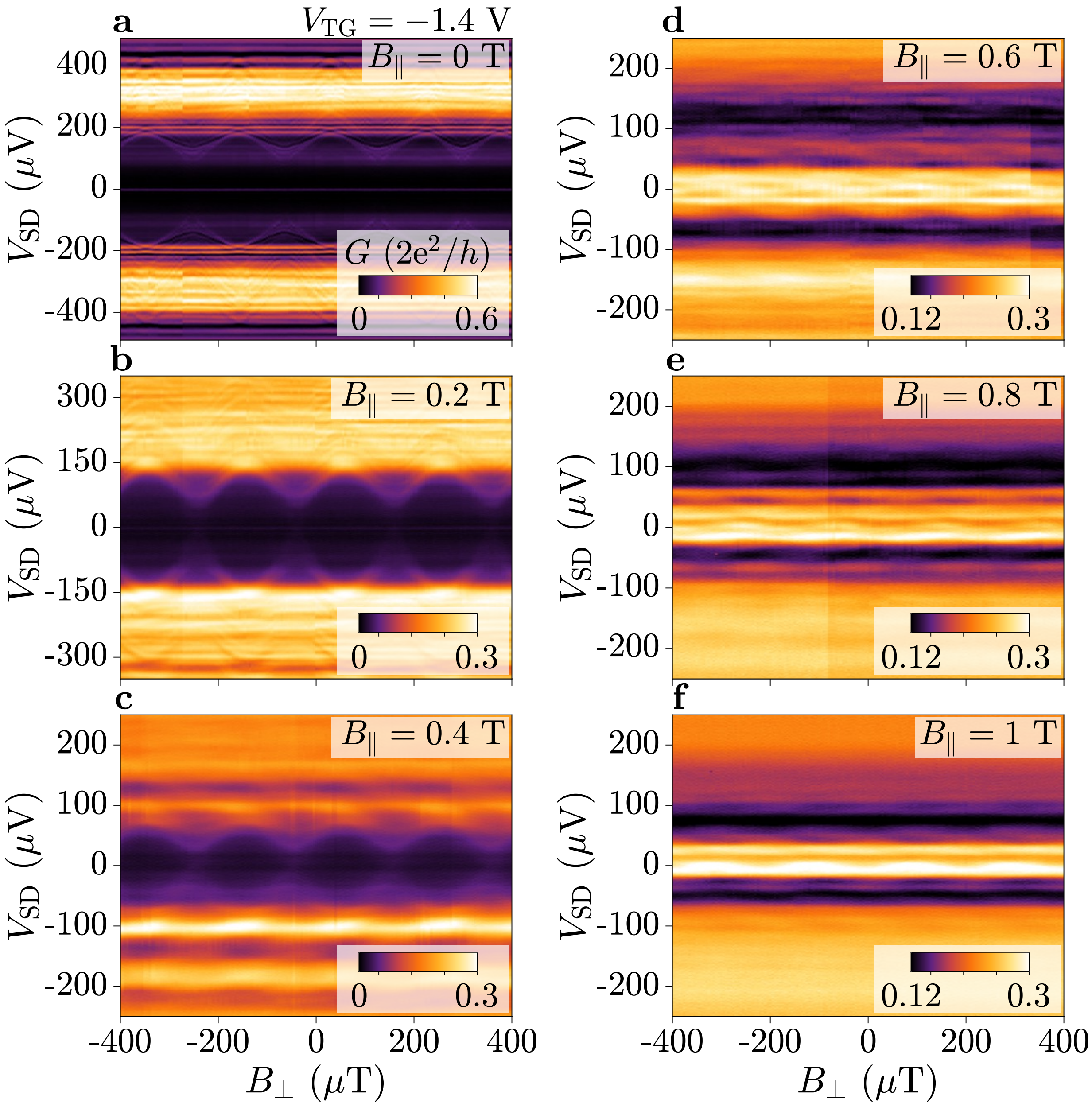


Figure S.9: Differential conductance $G$ as a function of source-drain bias $V_{SD}$ and perpendicular magnetic field $B_\perp$, for different values of in-plane magnetic field $B_\parallel$. Taken at a top-gate voltage of $V_{TG} = -1.4$ V and tunnel-gate voltages $(V_{T,L}, V_{T,R}) = (-1.835, -1.805)$ V.

contrast, only few modes were visible at $V_{\mathrm{TG}} = -1.4$ V [Fig. S.9(a)]. No $B_{\perp}$-dependent conductance features were observed for top-gate voltages $V_{\mathrm{TG}} < -1.4$ V. For increasing in-plane magnetic field $B_{\parallel}$, superconductivity in the tunnel probe was suppressed [Figs. S.8(b) and S.9(b)] and $B_{\perp}$-dependent conductance features moved closer to $V_{\mathrm{SD}} = 0$ [Figs. S.8(c) and S.9(c)]. At $B_{\parallel} = 0.6$ T, the superconducting gap was suppressed at both top-gate voltages and conductance features had very weak $B_{\perp}$-dependence close to $V_{\mathrm{SD}} = 0$ [Figs. S.8(d) and S.9(d)]. For larger in-plane fields, some phase-dependence appeared to recover although this was difficult to distinguish due to the poor visibility of conductance features corresponding to individual ABSs [Figs. S.8(e, f) and S.9(e, f)].

The superconducting gap was suppressed at $B_{\parallel} = 0.6$ T at all measured top-gate voltages. This is consistent with current-biased measurements [see Fig. 1(e) of the Main Text], where the minimum in the switching current occurred at $B_{\parallel} = 0.6$ T independent of top-gate voltage $V_{\mathrm{TG}}$. These results suggest that the cause of gap closure is independent of the properties of the normal region of the junction. Since orbital effects depend only on the properties of the superconducting leads, these findings are consistent with gap closure induced by orbital effects.

## 8 Tunneling Spectroscopy in Device 5

Tunneling spectroscopy was performed in an additional device to those shown in the Main Text, which was identical to Device 1 in all aspects other than the length of the superconducting leads $L_{\mathrm{SC}} = 400$ nm. The superconducting loop in this device, Device 5, was identical to that of Device 2 [Figs. 3(a, b) of the Main Text], where the switching current was measured. Conductance maps for different values of in-plane magnetic field $B_{\parallel}$ are shown in Figs. S.10 and S.11, for $V_{\mathrm{TG}} = 0.8$ V and $V_{\mathrm{TG}} = 0.2$ V respectively. These each correspond to the situation of a large [Fig. S.10(a)] or small [Fig. S.11(a)] number of modes, similar to Figs. S.8 and S.9 for Device 1. On increasing $B_{\parallel}$, the superconducting gap in

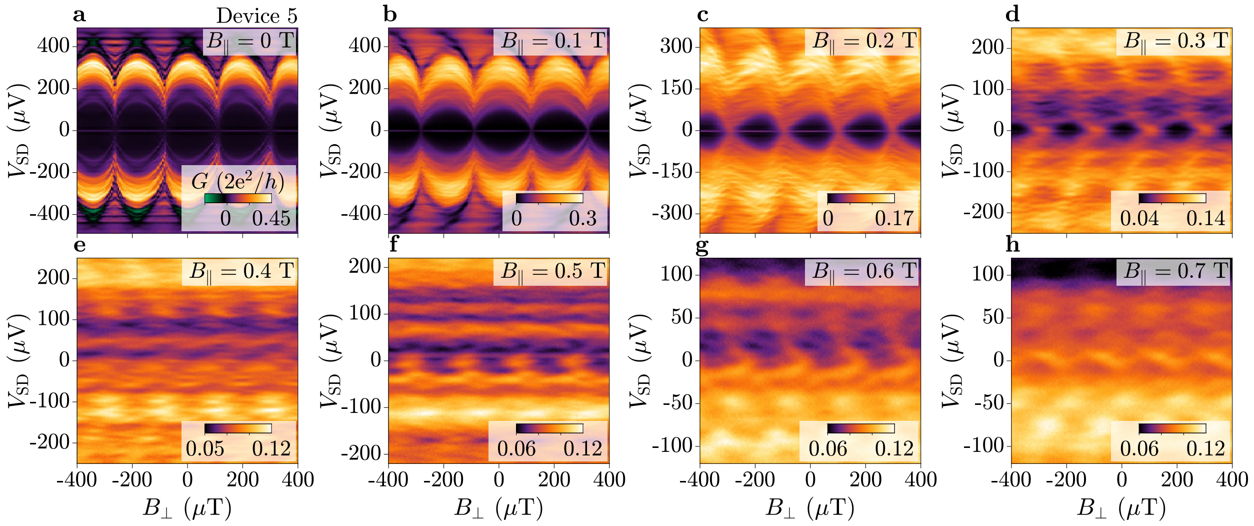


Figure S.10: Differential conducance $G$ of Device 5, which was identical to Device 1 other than the superconducting lead length, which was $L_{SC} = 400$ nm. Conductance maps for different in-plane magnetic fields $B_\parallel$, taken at a top-gate voltage $V_{TG} = 0.8$ V.

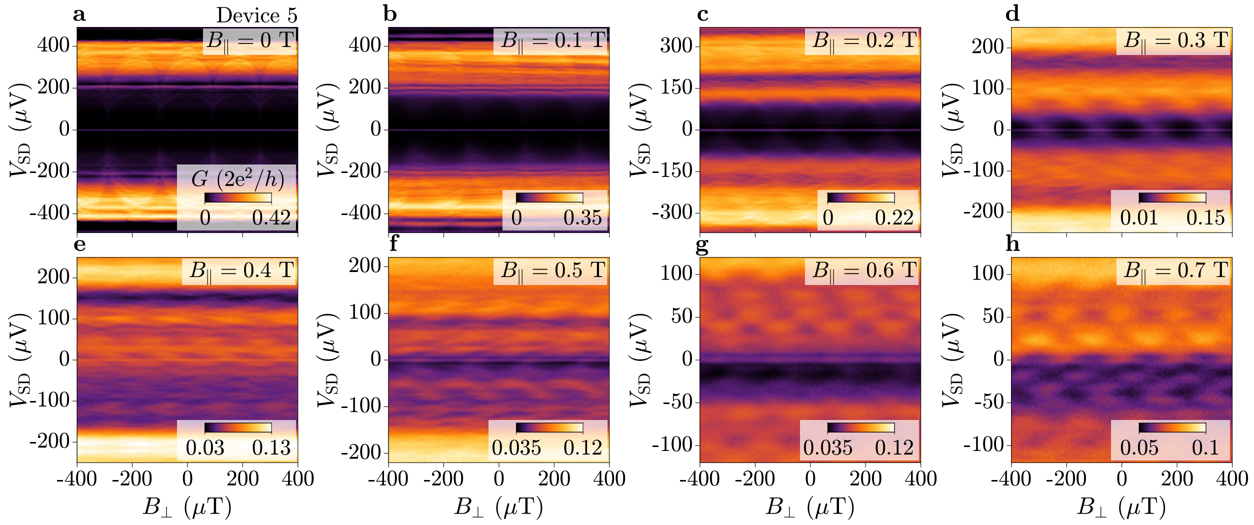


Figure S.11: Differential conducance $G$ of Device 5 at different in-plane magnetic fields $B_\parallel$, for $V_{TG} = 0.2$ V.

the tunnel probe was softened [Figs. S.10(b) and S.11(b)] and conductance features moved closer to $V_{\mathrm{SD}} = 0$ [Figs. S.10(c, d) and S.11(c, d)] until the gap between conductance features was closed at $B_{\parallel} = 0.4$ T [Figs. S.10(e) and S.11(e)]. For larger $B_{\parallel}$, the gap between conductance features reopened and there was a stronger $B_{\perp}$-dependence [Figs. S.10(f, g) and S.11(f, g)]. At $B_{\parallel} = 0.7$ T, the gap closed again and superconducting features were suppressed [Figs. S.10(h) and S.11(h)].

Closure of the superconducting gap was shown to occur at $B_{\parallel} = 0.4$ T in Device 5, for two top-gate voltages. This is consistent with the minimum in the switching current of Device 2, which had an identical SQUID loop, Al constriction and SNS junction. Tunneling spectroscopy showed a reopening of the gap between conductance features at larger in-plane fields, where a reentrant supercurrent was measured in current-biased experiments. The closure of the superconducting gap and minimum in the switching current both occurred at $B_{\parallel} \approx 0.4$ T, at multiple top-gate voltages with an uncertainty of $< 0.1$ T. This is significantly lower than the value found for Device 1, and consistent with the expected in-plane field at which one flux quantum threads the area underneath the superconducting leads. This supports the conclusion that gap closure in these devices is induced by orbital effects in the superconducting leads.

# 9 Devices with Varying Superconducting Lead Length

Measurements were performed on devices with varying superconducting lead length $L_{\mathrm{SC}}$ [see Fig. 2 of the Main Text]. Devices consisted of a superconducting loop identical to that of Device 1, other than the length of the superconducting lead which had values $L_{\mathrm{SC}} = 400$ nm, 350 nm and 180 nm for Devices 2-4 respectively. These devices did not have a tunnel probe proximal to the SNS junction, so only current-biased measurements were possible. Each device had two gates: a top-gate $V_{\mathrm{TG}}$ identical to that of Device 1 to tune the charge density in the SNS junction; and a global gate covering the exposed InAs regions around the junction

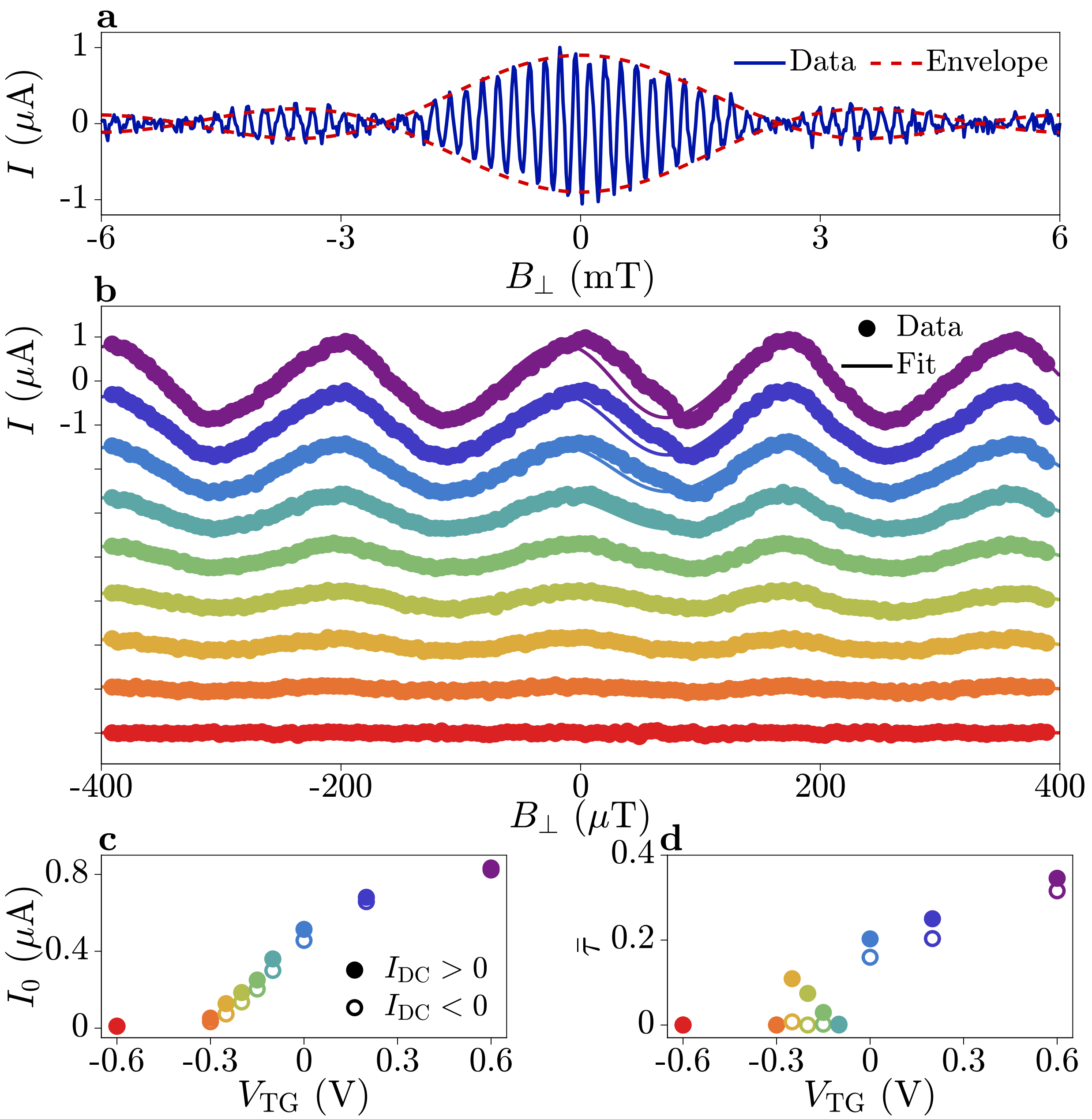


Figure S.12: (a) Switching current $I$ of Device 2 as a function of perpendicular magnetic field $B_\perp$, across a wide range of $\pm 6$ mT. Data (blue solid line) is fitted with an envelope function (red dashed line) of a Fraunhofer interference pattern. (b) Switching current as a function of perpendicular magnetic field $B_\perp$, after subtracting the background corresponding to the Al constriction. The background is determined from $V_{TG} = -0.6$ V (red circles), where the planar junction is considered to be completely closed since no oscillations in switching current were observed. Data at different top-gate voltages (circles) are fitted with a formula for the current-phase relation of Andreev bound states (line), at each top-gate voltage $V_{TG}$ denoted by the color [defined in (c)]. The fit incorporates the results obtained for the envelope in (a). Each trace is offset by 1 $\mu$A. (c, d) Results of the fit presented in (b): maximum switching current $I_C$ and transmission $\bar{\tau}$, for (c) and (d) respectively. Results for positive (negative) applied current $I_{DC}$ plotted as full (empty) markers.

and superconducting loop. The global gate was set to $V_{\text{Global}} < -1.5$ V throughout the experiment, such that the exposed InAs was depleted everywhere other than in the junction region.

Switching current measurements were performed for increasing in-plane magnetic field $B_{\parallel}$. At each value of $B_{\parallel}$, the switching current was first measured across a wide range of $B_{\perp}$ at the most positive top-gate voltage. After subtracting a slowly varying background corresponding to the Al constriction, a recognisable Fraunhofer interference pattern was observed [Fig. S.12(a), blue line]. In Devices 2 and 3, where the superconducting leads were large, flux focusing effects were strong. This caused a minimum in the Fraunhofer interference pattern at relatively small perpendicular fields $B_{\perp}$. It was therefore important to consider the envelope of switching current oscillations due to Fraunhofer interference. This was extracted from the data by filtering out the high frequency oscillatory component, and fitting the result with the following equation

$$I(B_{\perp}) = I_0 \left| \text{sinc}\left( \frac{B_{\perp} - B_0^{(\text{env})}}{B_{\text{min.}}} \right) \right| \tag{S.3}$$

There were three free parameters: the maximum current $I_0$, the perpendicular field at which the current was maximum $B_0^{(\text{env})}$ and the perpendicular field at which the first minimum occurred $B_{\text{min.}}$. The result of this fit for the data in Fig. S.12(a) is shown as the dashed red line. The in-plane field was aligned such that the maximum of the Fraunhofer pattern was close to $B_{\perp} = 0$ for each value of in-plane field. This was different in each device, due to flux focusing effects, so a different alignment was needed for each device. As such, the Reference Device was measured with each field alignment, to make a direct comparison.

At a given in-plane magnetic field, the switching current was measured as a function of perpendicular magnetic field $B_{\perp}$ for different top-gate voltages $V_{\text{TG}}$. The most negative top-gate voltage was chosen such that no oscillations were visible, where the SNS junction is assumed to be completely closed. The bias current therefore only flowed through the Al constriction, giving a direct evaluation of the switching current of the constriction as a

function of $B_\perp$. This background switching current was subtracted from the data at other $V_{\mathrm{TG}}$, to obtain the current-phase relation at each top-gate voltage [see Fig. S.12(b)]. The data (circles) for each $V_{\mathrm{TG}}$ [colors, defined in (c)] was fitted with Eq. S.2, adjusted to account for the envelope given by Eq. S.3:

$$I(B_\perp) = I_0 \left| \mathrm{sinc}\left( \frac{B_\perp - B_0^{(\mathrm{env})}}{B_{\mathrm{min.}}} \right) \right| \cdot \frac{\bar{\tau} \sin\left[ 2\pi \frac{(B_\perp - B_0)A}{\Phi_0} \right]}{E_{\mathrm{A}} \left[ 2\pi \frac{(B_\perp - B_0)A}{\Phi_0} \right] / \Delta} \tag{S.4}$$

Equation S.4 takes the fixed parameters $B_0^{(\mathrm{env})}$ and $B_{\mathrm{min.}}$ obtained from the fit to Eq. S.3. There are therefore only three free parameters, as in Eq. S.2: $I_0$, $\bar{\tau}$ and $B_0$. As for Device 1, $I_0$ is calculated as the maximum $I(B_\perp)$. The fit for the data in Fig. S.12(b) is shown as the colored lines, with the results for $I_0$ and $\bar{\tau}$ in (c) and (d) respectively [positive (negative) bias currents are indicated by the full (empty) markers]. This procedure is applied to every switching current measurement for Devices 2-4, to obtain the values shown in Fig. 2 of the Main Text. Measurements for positive and negative $B_\parallel$ are combined using the same method as for Device 1, as described above.

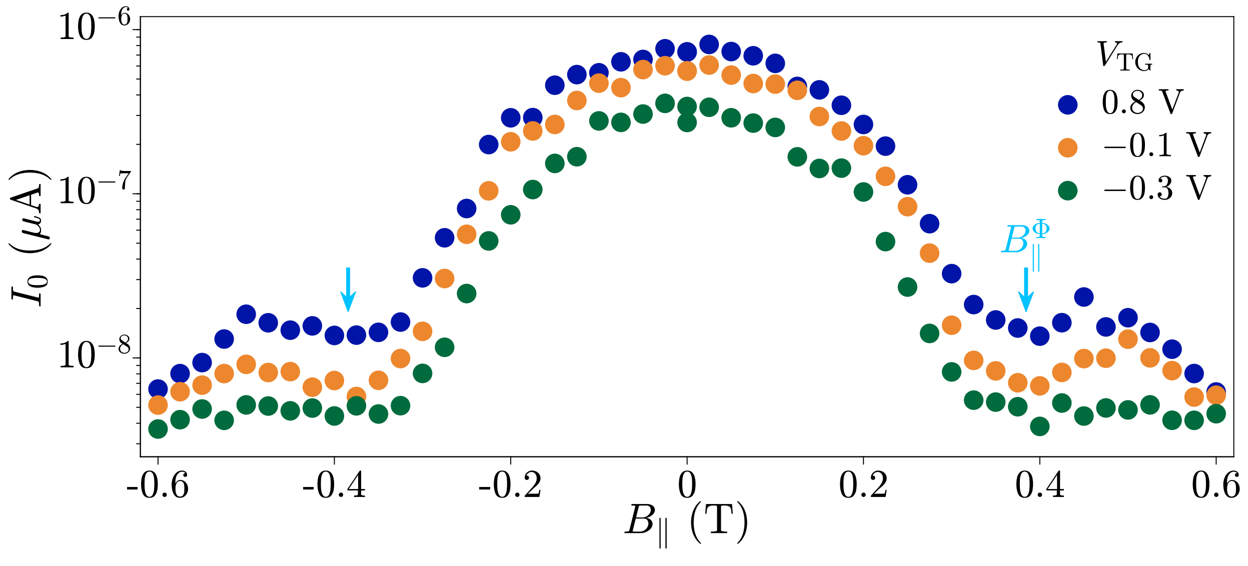


Figure S.13: Maximum switching current of Device 2 ($L_{\mathrm{SC}}$ = 400 nm) as a function of in-plane magnetic field $B_\parallel$, for different top-gate voltages $V_{\mathrm{TG}}$ (indicated by colors). Minimum in switching current at $|B_\parallel^\Phi| = 0.38 \pm 0.02$ T is indicated by the turquoise arrows.

Figure S.13 shows the maximum switching current $I_0$ in Device 2, as a function of in-plane magnetic field $B_{\parallel}$, for different top-gate voltages $V_{\mathrm{TG}}$. Measurements were performed for positive current bias $I_{\mathrm{DC}} > 0$, sweeping away from $B_{\parallel} = 0$ in both positive and negative field directions. The maximum switching current is obtained from SQUID oscillations as a function of perpendicular field $B_{\perp}$. A minimum and increase in the switching current occurred at $|B_{\parallel}| \equiv B_{\parallel}^{\Phi} = 0.38 \pm 0.02$ T for all $V_{\mathrm{TG}}$ [turquoise arrows]. This result is consistent with that of Fig. 2 of the Main Text, and one flux quantum threading the area underneath the superconducting leads. Two flux quanta are expected to thread the area underneath the superconducting leads at $|B_{\parallel}| = 2B_{\parallel}^{\Phi} \approx 0.8$ T. Although measurements at $|B_{\parallel}| > 0.6$ T were not possible, due to a large decrease in the switching current of the Al constriction at large fields, the trend in Fig. S.13 suggests a second minimum at larger $B_{\parallel}$.

## 10 Type A Phase Shifts in the Current Phase Relation

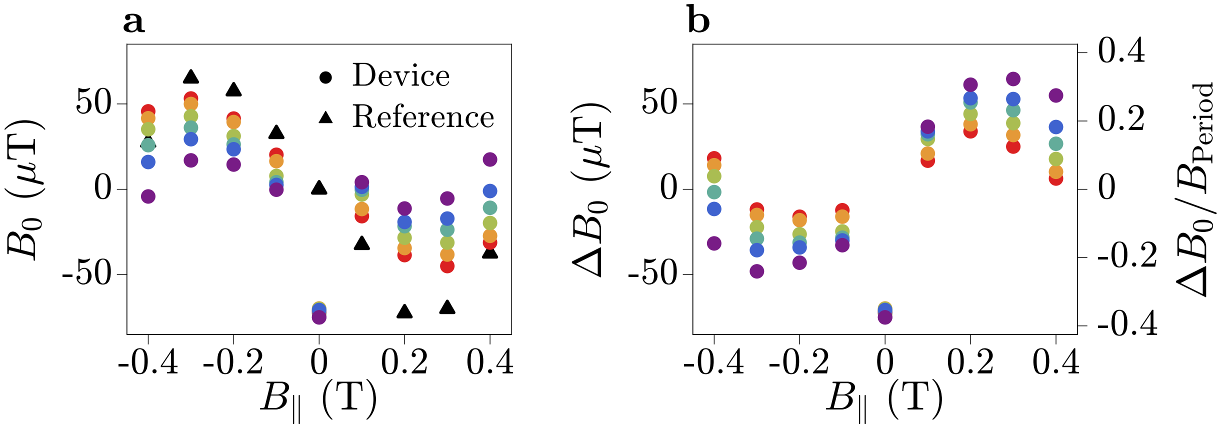


Figure S.14: (a) Perpendicular field shift $B_0$ in Device 1 [circles] and Reference Device [triangles], as in Fig. 1(f) of the Main Text zoomed-in close to $B_{\parallel} = 0$. (b) Magnetic field shift $\Delta B_0$ of the data in (a), relative to the Reference Device.

Type A shifts at small in-plane magnetic fields were quantified by comparing with the most negative top-gate voltage measured in that device. Figure S.14(a) shows a zoom-in of Fig. 1(f) of the Main Text, close to $B_{\parallel} = 0$. The magnetic field shift $\Delta B_0$ is shown in

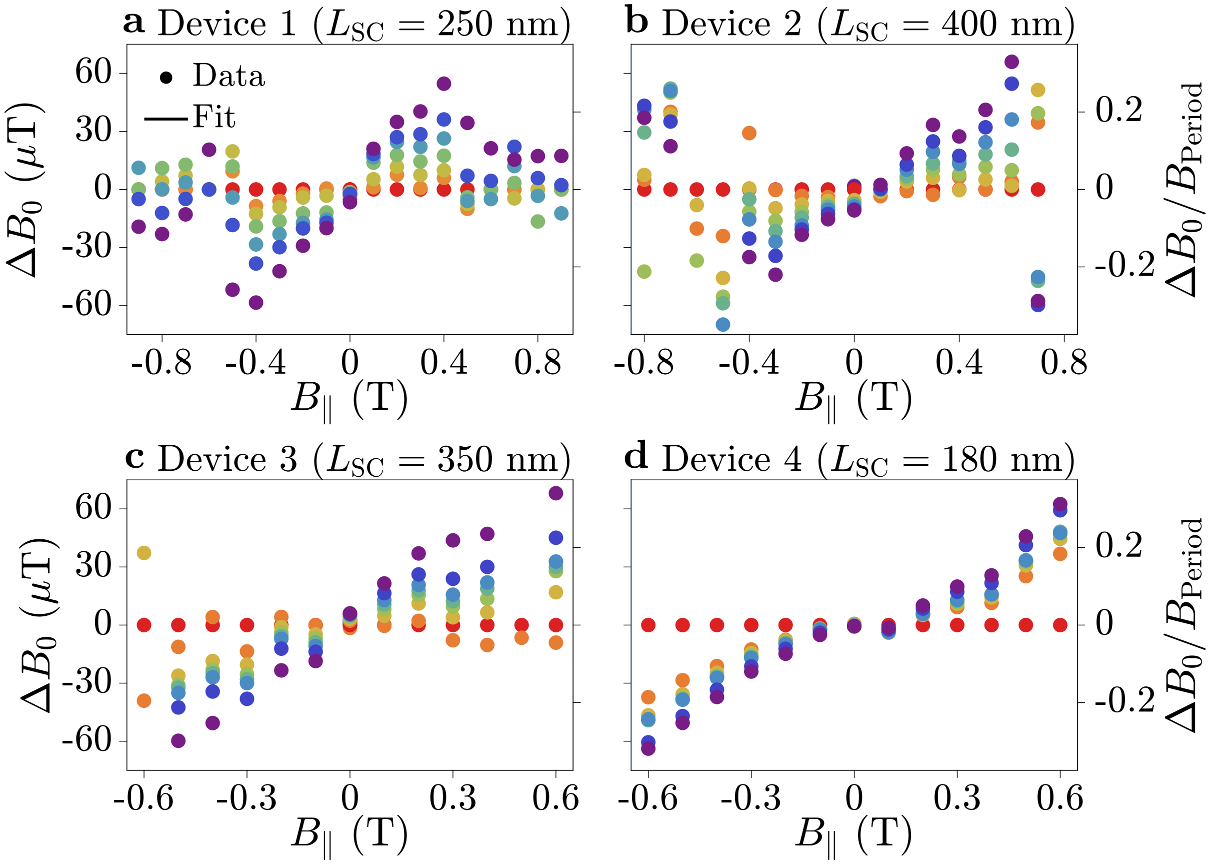


Figure S.15: Perpendicular field offset $\Delta B_0$ relative to the most negative top-gate voltage, as a function of in-plane magnetic field $B_{\parallel}$ for different top-gate voltages $V_{\mathrm{TG}}$ [indicated by the color, defined in Fig. S.16(e-h)], for Devices 1–4 respectively. Data is plotted for the full range of $B_{\parallel}$ measured in each device.

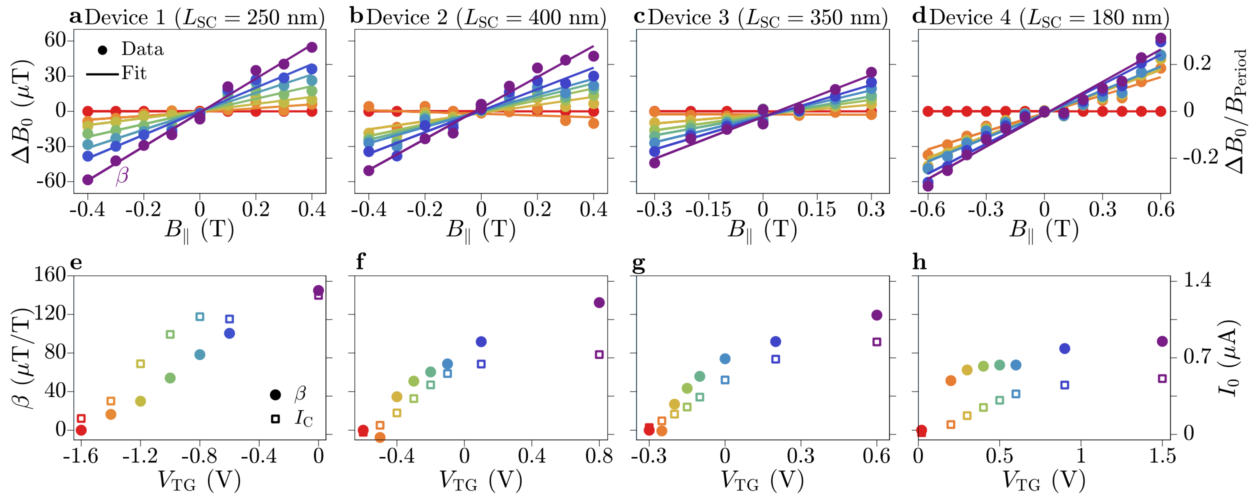


Figure S.16: (a-d) Perpendicular field offset $\Delta B_0$ for Devices 1–4, as in Fig. S.15, plotted in the range $|B_\parallel| \lesssim B_\parallel^\Phi$. Data (circles) is fitted with a linear curve at each $V_{TG}$ (lines), giving the gradient $\beta$. (e-h) Gradient $\beta$ extracted from (a-d) plotted as a function of top-gate voltage (filled circles, left axis). The maximum switching current as a function of top-gate voltage is also plotted for each Device (empty squares, right axis).

Fig. S.14(b), where in this case the values of $B_0$ in Device 1 are plotted relative to those of the Reference Device [triangles in Fig. S.14(a)]. The trend in $\Delta B_0$ is unexpected, and seems to originate from a systematic difference between the $B_\parallel$-dependence of Device 1 and the Reference Device. The trend is similar to the "S"-shape of $B_0$ at small $B_\parallel$, which was attributed to flux focusing effects in the region surrounding each device.[3] We therefore consider that small differences between the geometry of the Reference Device and Device 1 give rise to slightly different levels of flux focusing, which are particularly pronounced at small in-plane fields. Phase shifts must therefore be compared within the same device. The most negative top-gate voltage is taken as a reference point in this case, since it corresponds to the smallest switching current, where both the electron sheet density and the spin-orbit coupling strength $\alpha$ are expected to be small.[13] From Ref.,[13] we estimate $\alpha < 20$ meVÅ, approximately 10% of its value at high density (corresponding to $V_{TG} \sim 0$). This approach was recently employed in a related work.[14]

Gate-dependent Type A phase shifts were observed in all devices, across the full range of in-plane fields $B_\parallel$ measured in each device [see Fig. S.15]. However, there is a discontinuity

in the phase shift due to Type B shifts for $|B_{\|}| \gtrsim B_{\|}^{\Phi}$, where $B_{\|}^{\Phi}$ is the field at which the superconducting gap is suppressed by orbital effects. In addition, the small switching current measured at large in-plane fields makes analysis challenging, leading to less reliable estimates of $\Delta B_0$ values at large $B_{\|}$. As a result, quantitative analysis is performed for small in-plane fields, where only Type A shifts were present. The results for Devices 1–4 are summarized in Fig. S.16. The perpendicular field offset relative to the most negative gate voltage, $\Delta B_0$, was linear with in-plane field $B_{\|}$ with steeper gradient $\beta$ for more positive top-gate voltage $V_{\mathrm{TG}}$ [Figs. S.16(a-d), colors defined in (e-h)]. The data (circles) are fitted with a linear curve (lines) to extract the gradient $\beta$, which is plotted in Figs. S.16(e-h) (filled circles) for Devices 1-4 respectively. The maximum switching current $I_0$ at $B_{\|} = 0$ is also plotted as a function of top-gate voltage $V_{\mathrm{TG}}$ (empty squares). The trend of $\beta$ with $V_{\mathrm{TG}}$ is similar to that of the maximum switching current $I_0$.

At the maximum $V_{\mathrm{TG}}$, where $I_0$ was large, $\beta \gtrsim 100$ $\mu$T/T for all devices independent of the superconducting lead length $L_{\mathrm{SC}}$. The size of the shift $\Delta B_0$ did not depend strongly on the switching current at that in-plane field, rather on the switching current at $B_{\|} = 0$. This is because the switching current at an in-plane field is significantly influenced by orbital effects, independent of the carrier density at that top-gate voltage. The maximum switching current is linked to the carrier density in the junction, since at lower densities there are fewer transverse modes to carry the supercurrent.[15] The switching current is therefore indicative of the carrier density in the InAs, despite that the gate voltages might differ between devices due to local disorder, inhomogeneous material properties and fabrication imperfections. For decreasing $V_{\mathrm{TG}}$, the carrier density decreases causing both $I_0$ and $\beta$ to decrease [Figs. S.16(e-h)]. This follows a trend consistent with that of Ref.,[13] which directly measured the spin-orbit coupling strength as a function of carrier density, in similar InAs quantum wells.

However, the size of these Type A phase shifts is much larger than would be expected for a single ballistic channel,[5,16] using typical values for the spin-orbit coupling strength $\alpha$ of InAs.[13] For $\alpha \approx 100$ meVÅ and $v_{\mathrm{F}} \approx 1 \cdot 10^6$ ms$^{-1}$, we estimate the gradient of magnetic

fields shifts to be $\beta \sim 0.2\ \mu$T/T, orders of magnitude smaller than the experimentally obtained values. Similar observations were made in Ref.,[14] where anomalous phase shifts were reported for planar Josephson junctions in InAs/Al heterostructures. The anomalous phase shift was shown to be consistent with that of ABSs in tunneling spectroscopy [Fig. 4 of the Main Text], implying that the phase shift was not dominated by low transmission modes but had contributions from all modes in the junction.

# 11 Type A Phase Shifts in Tunneling Spectroscopy

Figure 4 shows differential conductance maps for different top-gate voltages $V_{\mathrm{TG}}$. The perpendicular field at which the ABS energy was lowest was taken to be where the partial derivative of the differential conductance with respect to perpendicular field, $\partial G/\partial B_{\perp}$, was zero at a fixed source-drain bias $V_{\mathrm{SD}}$. The closest conductance feature to $V_{\mathrm{SD}} = 0$ was considered. This procedure was repeated across 5 lobes, for positive and negative bias, and extracted values of $B_{\perp}$ were shifted by integer multiples of the period $B_{\mathrm{Period}}$ to give values within $[-B_{\mathrm{Period}}/2, B_{\mathrm{Period}}/2]$. A similar procedure was followed by considering the position where the conductance was closest to $V_{\mathrm{SD}} = 0$, which corresponds to $\varphi \approx \pi$. All methods gave a similar trend and similar quantitative values for the phase shift. The data plotted in Fig. 4(i) of the Main Text is the average of all values obtained from these methods, with the error bars giving the standard deviation.

# 12 Phase Shifts due to Kinetic Inductance of the Superconducting Loop

Switching current measurements were performed by applying large bias currents to the SQUID device. Since the epitaxial Al is very thin, it has an appreciable kinetic inductance $L_{\mathrm{K}}$, which generates a flux $\Phi_{\mathrm{K}} = L_{\mathrm{K}}(I_{\mathrm{cons.}} - I_{\mathrm{SNS}})/2$, where $I_{\mathrm{cons.}}$ and $I_{\mathrm{SNS}}$ are the currents

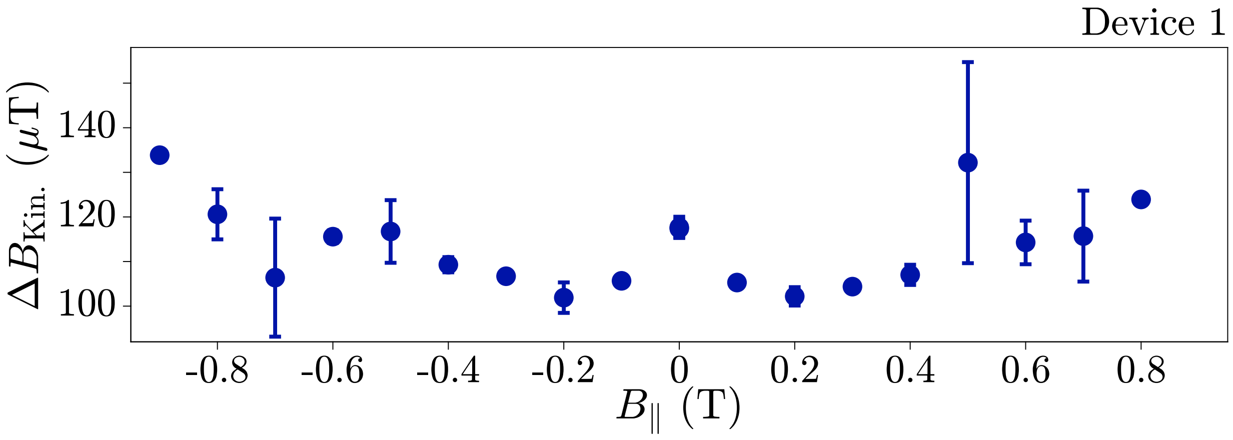


Figure S.17: Shift in perpendicular field between current-phase relation traces measured with positive and negative bias currents, $\Delta B_{\mathrm{Kin.}}$. Points are plotted as an average over all top-gate voltages $V_{\mathrm{TG}}$, with errorbars indicating the standard deviation of all $V_{\mathrm{TG}}$ values.

flowing in the Al constriction and SNS junction, respectively. The kinetic inductance of the loop is estimated as[17]

$$L_{\mathrm{K}} = N_{\square}\frac{h}{2\pi^2}\frac{R_{\square}}{\Delta} \approx 66 \text{ pH}, \tag{S.5}$$

where $N_{\square} = 38$ is the number of squares in the superconducting loop, $R_{\square} \approx 1.5\ \Omega$ is the normal-state sheet resistance per unit square measured in a Hall bar geometry on the same material, and $\Delta \approx 180\ \mu$eV is the superconducting gap of Al. This gives a shift of $\Delta B_{\mathrm{Kin.}} \approx 110\ \mu$T, for typical currents $(I_{\mathrm{cons.}} - I_{\mathrm{SNS}})$ in the SQUID loop. The shift $\Delta B_{\mathrm{Kin.}}$ between positive and negative currents is shown in Fig. S.17. No top-gate dependence was observed, so points were averaged over all top-gate voltages. The field shift $\Delta B_{\mathrm{Kin.}}$ increased for increasing magnitude of in-plane magnetic field, consistent with an increasing kinetic inductance due to quasiparticle generation in the superconducting loop. The values of $\Delta B_{\mathrm{Kin.}}$ in Fig. S.17 are consistent with the field shift estimated from the kinetic inductance in Eq. S.5.

## 13 Data Availability

The data that support the findings of this study are available upon reasonable request from the corresponding author.

## References


(1) Peltonen, J. T.; Muhonen, J. T.; Meschke, M.; Kopnin, N. B.; Pekola, J. P. Magnetic-Field-Induced Stabilization of Nonequilibrium Superconductivity in a Normal-Metal/Insulator/Superconductor Junction. *Phys. Rev. B* **2011**, *84*, 220502.

(2) Chen, Y.; Lin, Y.-H.; Snyder, S. D.; Goldman, A. M. Stabilization of Superconductivity by Magnetic Field in Out-of-Equilibrium Nanowires. *Phys. Rev. B* **2011**, *83*, 054505.

(3) Suominen, H. J.; Danon, J.; Kjaergaard, M.; Flensberg, K.; Shabani, J.; Palmstrøm, C. J.; Nichele, F.; Marcus, C. M. Anomalous Fraunhofer Interference in Epitaxial Superconductor-Semiconductor Josephson Junctions. *Phys. Rev. B* **2017**, *95*, 035307.

(4) Beenakker, C. W. J.; van Houten, H. Josephson Current Through a Superconducting Quantum Point Contact Shorter than the Coherence Length. *Phys. Rev. Lett.* **1991**, *66*, 3056–3059.

(5) Yokoyama, T.; Eto, M.; Nazarov, Y. V. Anomalous Josephson Effect Induced by Spin-Orbit Interaction and Zeeman Effect in Semiconductor Nanowires. *Phys. Rev. B* **2014**, *89*, 195407.

(6) Haxell, D. Z.; Cheah, E.; Křížek, F.; Schott, R.; Ritter, M. F.; Hinderling, M.; Belzig, W.; Bruder, C.; Wegscheider, W.; Riel, H.; Nichele, F. Measurements of Phase Dynamics in Planar Josephson Junctions and SQUIDs. *Phys. Rev. Lett.* **2023**, *130*, 087002.

(7) Pientka, F.; Keselman, A.; Berg, E.; Yacoby, A.; Stern, A.; Halperin, B. I. Topological Superconductivity in a Planar Josephson Junction. *Phys. Rev. X* **2017**, *7*, 021032.

(8) Shabani, J.; Kjaergaard, M.; Suominen, H. J.; Kim, Y.; Nichele, F.; Pakrouski, K.; Stankevic, T.; Lutchyn, R. M.; Krogstrup, P.; Feidenhans'l, R.; Kraemer, S.; Nayak, C.; Troyer, M.; Marcus, C. M.; Palmstrøm, C. J. Two-Dimensional Epitaxial Superconductor-Semiconductor Heterostructures: A Platform for Topological Superconducting Networks. *Phys. Rev. B* **2016**, *93*, 155402.

(9) Winkler, R. *Spin-orbit Coupling Effects in Two-Dimensional Electron and Hole Systems*; Springer Berlin Heidelberg, 2003.

(10) Dartiailh, M. C.; Mayer, W.; Yuan, J.; Wickramasinghe, K. S.; Matos-Abiague, A.; Žutić, I.; Shabani, J. Phase Signature of Topological Transition in Josephson Junctions. *Phys. Rev. Lett.* **2021**, *126*, 036802.

(11) Fornieri, A. et al. Evidence of Topological Superconductivity in Planar Josephson Junctions. *Nature* **2019**, *569*, 89–92.

(12) Ren, H.; Pientka, F.; Hart, S.; Pierce, A. T.; Kosowsky, M.; Lunczer, L.; Schlereth, R.; Scharf, B.; Hankiewicz, E. M.; Molenkamp, L. W.; Halperin, B. I.; Yacoby, A. Topological Superconductivity in a Phase-Controlled Josephson Junction. *Nature* **2019**, 3–8.

(13) Wickramasinghe, K. S.; Mayer, W.; Yuan, J.; Nguyen, T.; Jiao, L.; Manucharyan, V.; Shabani, J. Transport Properties of Near Surface InAs Two-Dimensional Heterostructures. *Appl. Phys. Lett.* **2018**, *113*, 262104.

(14) Mayer, W.; Dartiailh, M. C.; Yuan, J.; Wickramasinghe, K. S.; Rossi, E.; Shabani, J. Gate Controlled Anomalous Phase Shift in Al/InAs Josephson Junctions. *Nat. Commun.* **2020**, *11*, 212.

(15) Kjaergaard, M.; Suominen, H. J.; Nowak, M. P.; Akhmerov, A. R.; Shabani, J.; Palmstrøm, C. J.; Nichele, F.; Marcus, C. M. Transparent Semiconductor-Superconductor Interface and Induced Gap in an Epitaxial Heterostructure Josephson Junction. *Phys. Rev. Appl.* **2017**, *7*, 034029.

(16) Buzdin, A. Direct Coupling Between Magnetism and Superconducting Current in the Josephson $\varphi_0$ Junction. *Phys. Rev. Lett.* **2008**, *101*, 107005.

(17) Annunziata, A. J.; Santavicca, D. F.; Frunzio, L.; Catelani, G.; Rooks, M. J.; Frydman, A.; Prober, D. E. Tunable Superconducting Nanoinductors. *Nanotechnology* **2010**, *21*, 445202.
